# Evaluation of large Language models on pediatric asthma: a comparative study of Claude3-Opus, Gemini 2.0, ChatGPT-4o, and DeepSeek—a cross-sectional questionnaire study

**DOI:** 10.1186/s12911-026-03371-x

**Published:** 2026-02-10

**Authors:** Ying-qi Hang, Jie Wu, Li Bai, Mingyun Wu, Jianer Yu, Liang Li, Xiang Piao

**Affiliations:** 1 Shanghai Municipal Hospital of Traditional Chinese Medicine, Shanghai University of Traditional Chinese Medicine, Shanghai, China; 2 Shanghai University of Traditional Chinese Medicine, Shanghai, China; 3 Ankang Hospital of Traditional Chinese Medicine/the Seventh Clinical Medical School, Shaanxi University of Chinese Medicine, Shaanxi, China

**Keywords:** Artificial intelligence, ChatGPT, Claude, Gemini, DeepSeek, Pediatric asthma

## Abstract

**Background:**

Artificial intelligence (AI) has shown potential for enhancing medical practice and improving patient outcomes. However, the efficacy and linguistic accessibility of Large Language Models(LLMs) in pediatric asthma management remain underexplored. This study evaluated the performance of four LLMs in generating clinical information within this domains.

**Methods:**

We administrated 15 guideline-based pediatric asthma inquiries to hatGPT-4o, Claude 3 Opus, Gemini 2.0, and DeepSeek. Anonymized responses were independently evaluated by three board-certified pediatric pulmonologists using DISCERN instrument (score range 16–80). Readability was assessed using six standard indices. Inter-rater reliability was measured with intraclass correlation coefficients (ICC). Statistical analysis included repeated measures and post-hoc comparisons with effect size reporting.

**Results:**

No significant difference was found in the overall quality of health information (DISCERN scores) among the four LLMs (F(3,56) = 0.144, *p* =.933, η² =0.008), with all mean scores clustered within a narrow “fair-to-good” range (50.3–51.9). However, significant differences were observed in readability: ChatGPT-4o generated significantly more comprehensible text than DeepSeek (FRE mean difference = 12.41, *p* =.005, Cohen’s d = 1.28), while DeepSeek performed significantly worse than all other models (all *p* <.05). Inter-rater reliability was high (ICC range: 0.849–0.901, all *p* <.001). Critically, the mean readability level of all outputs (FKGL: 13.2–14.9) far exceeded the recommended reading accessibility level for patient materials.

**Conclusions:**

While current LLMs can provide generally accurate information on pediatric asthma, their outputs exhibit significant limitations in readability for patient-facing use. ChatGPT‑4o shows relative advantages in comprehensibility, yet none meet recommended health-literacy standards. These findings underscore that AI should serve as a supplementary decision‑support tool under clinician supervision, not as a substitute for professional medical advice. Future work should prioritize the integration of adaptive text‑simplification features, validate AI‑generated content in real‑world clinical and caregiver settings, and expand evaluations to include emerging models and diverse chronic disease contexts.

**Supplementary Information:**

The online version contains supplementary material available at 10.1186/s12911-026-03371-x.

## Introduction

Artificial intelligence (AI), particularly large language models (LLMs) driven by natural language processing (NLP), holds potential for respiratory medicine, offering capabilities ranging from clinical decision support [1] to personalized patient education [2–3]. In adult respiratory care, NLP tools have demonstrated utility in interpreting asthma control metrics and synthesizing guideline-based recommendations, thereby optimizing clinical workflows [4–6]. However, the translation of these technologies into pediatric asthma management presents unique challenges [7]. Unlike adult care, pediatric management hinges on the health literacy of caregivers, necessitating continuous, clear communication to ensure treatment adherence and effective self-management [8].

Despite the proliferation of LLMs such as ChatGPT, Gemini, Claude, and DeepSeek, their practical utility in this high-stakes context remains constrained by a critical dual requirement [9]: factual precision and linguistic accessibility. While prior studies have scrutinized the accuracy of LLM-generated medical content [10], two significant gaps persist. First, there is a paucity of systematic evaluations using validated quality assessment instruments, such as DICERN [11], Second, perhaps more critically, the readability of these outputs, a key determinant of their safety and effectiveness for non-expert users-has been largely overlooked [12]. It remains unclear [13] whether current algorithms can bridge the gap between complex clinical guidelines and the lay language required for effective caregivers education [14–15].

This study aims to systematically evaluate the performance of four LLMs (ChatGPT-4o, Gemini 2.0, Claude 3 Opus, and DeepSeek) in addressing pediatric asthma management [16]. By employing validated instruments to assess informational quality (DISCERN) alongside multiple readability indices, we will analyze the responses to a standardized set of guideline-based clinical questions. The primary objective is to determine whether these models can generate outputs that are simultaneously clinically reliable and linguistically accessible to clinicians and patients/caregivers. This comparative analysis seeks to inform the safe and practical integration of LLMs into asthma care, particularly for enhancing patient education and supporting shared clinical decision-making.

## Methods

### Study design and model description

This cross-sectional comparative study evaluated the performance of four publicly available large language models (LLMs): ChatGPT-4o (OpenAI), Claude 3 Opus (Anthropic), Gemini 2.0 (Google DeepMind), and DeepSeek (DeepSeek AI). All queries were submitted through the models’ standard web interfaces. To ensure response independence and minimize contextual carryover, each question was posed within a newly initiated chat thread. To account for potential variability in input generation, all queries were submitted independently by three study investigators.

### Evaluators(participants)

Response quality was evaluated by an independent panel of three board-certified pediatric pulmonologists. Each expert possessed over ten years of clinical experience in pediatric respiratory practice and was blinded to the source (e.g., which AI model generated which response) during the scoring process. These specialists were not involved in the earlier stage of question development, thereby ensuring an unbiased assessment of response quality. Their primary role was to apply the DISCERN instrument to evaluate the accuracy, reliability, and comprehensiveness of each AI-generated answer.

### Development of the clinical question set

The study utilized 15 clinical questions (Supplementary file 1) derived from the 2024 Global Initiative for Asthma (GINA) Pediatric Guideline [16].The questions were stratified into two categories to reflect distinct end-user needs:

Caregiver/Patient-focused (*n* = 7): Targeted disease mechanisms, daily management, medication safety, and trigger avoidance (e.g., Questions 3, 4, 5, 9,10,11,12).

Clinician-focused (*n* = 8): Centered on guideline-based treatment strategies, step-up/step-down therapy, and exacerbation management (e.g., Questions 1,2,6,7,8,13,14,15).

Questions validity was established through a three-phase process: (1) Domain extraction from GINA chapters; (2) Question Drafting; (3) Expert Review and Finalization.

Domain Extraction: Key clinical topics were systematically mapped from core GINA chapters, including diagnosis, pharmacotherapy, exacerbation management, and prevention.

Question Drafting: Preliminary questions were formulated to reflect common clinical scenarios and typical patient/caregiver concerns within these domains.

Expert Review and Finalization: The independent pediatric specialists reviewed all drafted questions for clinical relevance, clarity, and the availability of a definitive evidence-based answer within the GINA framework. Final inclusion required consensus, resulting in the 15-question set.

### Outcome measures

#### Primary outcome: information quality (DISCERN)

The quality of response was assessed using the DISCERN instrument [17], a validated 16-item tool scoring from 1 (low quality) to 5 (high quality). Total score between 16 and 80, categorized as: Very Poor (16–26), Poor (27–38), Fair (39–50), Good (51–62), or Excellent (63–80). Three independent pediatric specialists, blinded to the AI model source, scored all responses. The mean score from the three raters for each question-model pair was used in the primary analysis.

#### Secondary outcome: text readability

Readability was assessed using six standardized indices calculated via the WebFX Readability Tool [18]: Flesch Reading Ease (FRE), Flesch-Kincaid Grade Level (FKGL), Gunning Fog Index (GFI), Simple Measure of Gobbledygook (SMOG), Coleman-Liau Index (CLI), and Automated Readability Index (ARI). Following American Medical Association (AMA) and National Institutes of Health (NIH) recommendations, a grade level ≤ 8 (or FRE ≥ 60) was considered appropriate for patient-facing materials. [19] (Table 1).

**Table 1:** Readability indices and their measurement frameworks

Readability Indices	Measurement regulations	Reading Level Interpretation
Flesch Kincaid Reading Ease(FRE)	Words per sentence and syllables per word	0–30: Very difficult; 31–60: Difficult; 61–70: Moderately easy; 71–100: Easy; Target for patient materials: ≥60
Flesch Kincaid Grade Level(FKGL)	Length of each sentence and syllables per sentence	US Grade levels^a^ (5th-grade to college graduate)
Gunning Fog Index(Gunning FOG)	Length, and complexity of each sentence	US Grade levels^a^ (5th-grade to college graduate)
SMOG Index(SMOG)	Complex word density of sentence	US Grade levels^a^ (5th-grade to college graduate)
Coleman Liau Index (CLI)	Number of characters and words per sentence	US Grade levels^a^ (5th-grade to college graduate)
Automated Readability Index(ARI)	Number of characters and length of each sentence	US Grade levels^a^ (5th-grade to college graduate)

^a^Each indice uses grade level as a unit of results

^b^The Flesch Kincaid Reading Ease utilizes a different scoring system, in which case the scale is from 0

### Statistical analysis

#### Power analysis

A power analysis was performed using G*Power 3.1. For a repeated-measures analysis of variance (ANOVA) with four group, assuming an alpha of 0.05, power of 0.80, and a moderate effect size (Cohen’s f = 0.30), a minimum of 12 question-answer pairs was required. This study utilized 15 pairs, providing adequate statistical power for the primary comparisons.

### Data analysis

Statistical analysis was performed using SPSS (version 28.0). Inter‑rater reliability for DISCERN scores was assessed using the intraclass correlation coefficient (ICC; two‑way random model). Normality was evaluated using the Shapiro-Wilk test. DISCERN scores and Readability analysis were compared using a one-way ANOVA. Post hoc comparisons were performed using the Bonferroni correction to adjust for multiple comparisons [20].

Effect sizes were reported as partial eta-squared (η²) for ANOVA, Cohen’s d for paired t-tests, and r (r = Z/√N) for Wilcoxon tests to facilitate interpretation of clinical significant. Statistical significance was set at *p* <.05 (two‑tailed), with Dunn–Bonferroni post‑ hoc tests for multiple comparisons if significant.

## Results

In our study, fifteen hypothetical asthma-related questions were delivered to the targeted AI tools, and the responses were rated by three independent experts using the DISCERN instrument to evaluate the overall quality of the health information. (Table 2: Chatgpt-4o as example). Further details on the performance of other AI tools are presented in Supplementary table 2S1.

**Table 2:** DISCERN analysis summary for AI tool’s asthma consultations.(Take ChatGPT as example)

ChatGPT-4o		DISCERN scores				DISCERN Category
		Specialist1	Specialist2	Specialist3	Mean Scores	
Question 1	What is the preferred initial treatment if a child has infrequent asthma symptoms.(e.g.1-2days/week or less)?	58	53	62	57.67	Good
Question 2	If I want to step down asthma treatment, how long should a child asthmatic symptoms be well controlled?	56	54	59	56.33	Good
Question 3	Can asthma be triggered by strong emotions or will the symptoms be worsened because of the unstable emotions?	55	54	51	53.33	Good
Question 4	During the asthma attack, do the air tubes collapse? Is More or less mucus produced in the air tubes?	52	50	56	52.67	Good
Question 5	By listening to the asthma’s patient chest with a stethoscope, Is a doctor manage to measure how bad asthma is?	53	51	53	52.33	Good
Question 6	What is the treatment option for children whose asthma is not adequately controlled by low-dose maintenance ICS-LABA with as-needed SABA?	45	49	50	48.00	Median
Question 7	List some add-on biologic therapy for children with uncontrolled severe asthma.	49	52	51	50.67	Good
Question 8	Will the risk of exacerbation increase when a child switch from Maintenance-and-reliever therapy(MART) to conventional lCS-LABA plus as-needed SABA?	49	48	52	49.67	Median
Question 9	Will a child be addicted to asthma medications if he uses them frequently?	46	44	48	46.00	Median
Question 10	After an asthmatic exacerbation, How soon should a review visit be scheduled? What does the frequency of visit depend on?	40	41	39	40.00	Median
Question 11	If a child is exposed to viral infections or seasonal allergen exposure, Is a short-term increase in maintenance lCS dose for 1–2 weeks be necessary?	55	52	50	52.33	Good
Question 12	When an a child with asthma is going to be exposed to something that triggers asthma, can he take medication just before exposure to prevent asthma?	60	58	59	59.00	Good
Question 13	For children aged 6–11,On what condition can the the treatment be successfully reduced?	63	61	57	60.33	Good
Question 14	Can high baseline of FeNO been used to predict exacerbation after step-down of lCS dose?	62	66	62	63.33	Excellent
Question 15	Before asthma treatment step-down, what should be evaluated by patients?	38	36	34	36.00	Bad
	Total DISCERN score (16–80)	*n* = 15				
	Very bad (16–26)	0				
	Bad (27–38)	1				
	Median (39–50)	4				
	Good (51–62)	9				
	Excellent (63–80)	1				
	Average score	51.87 ± 7.65				

Abbreviations: FeNO: fractional exhaled nitric oxide

### Quality of AI-generated responses

A one-way analysis of variance (ANOVA) revealed no statistically significant difference in the quality of health information, measured by DISCERN scores, among the four AI platforms: F(3, 56) = 0.144, *P* =.933. The effect size was negligible (η² = 0.008). Descriptive statistics indicated a narrow range of mean scores: ChatGPT-4o (51.87 ± 7.65), Gemini 2.0 (51.60 ± 9.04), DeepSeek (50.57 ± 6.40), and Claude3-Opus (50.33 ± 8.53) (Table 3).

**Table 3:** One-way-ANOVA result for DISCERN scores across AI platforms

	AI platforms				SS	df	MS	F	*P*	Effect Size (η²)
	Claude3- Opus (*n* = 15)	Gemini 2.0 (*n* = 15)	ChatGPT-4o (*n* = 15)	DeepSeek (*n* = 15)						
Disern scores (mean ± sd)	50.33 ± 8.53	51.60 ± 9.04	51.87 ± 7.65	50.57 ± 6.40	25.12	3	8.37	0.144	0.933	0.008

Abbreviations: SS = Sum of Squares, df = Degrees of Freedom, MS = Mean Square, F = F-value, p = P-value, η² = Eta-squared (effect size)

The inter-rater reliability among the three independent pediatric specialists has demonstrated high consistency. Intraclass correlation coefficients (ICCs) for the four platforms ranged from 0.849 to 0.901 (all *P* <.001), confirming high reliability in expert evaluation.(Table 4).

**Table 4:** Intraclass correlation

AI Platform	Rater	DISCERN Score (mean ± sd)	Median Score	ICC		Pfa
				*r*	*P* ^ICCb^	
Claude3-Opus	Specialist 1	50.2 ± 8.5	50	0.883	< 0.001	0.627
	Specialist 2	49.5 ± 9.2	51			
	Specialist 3	51.3 ± 7.4	54			
Gemini 2.0	Specialist 1	51.2 ± 8.9	51	0.9	< 0.001	0.165
	Specialist 2	50.7 ± 9.8	51			
	Specialist 3	52.9 ± 8.0	54			
ChatGPT-4o	Specialist 1	52.1 ± 7.6	53	0.901	< 0.001	0.408
	Specialist 2	51.3 ± 7.5	52			
	Specialist 3	52.2 ± 7.8	52			
DeepSeek	Specialist 1	49.6 ± 7.3	50	0.849	< 0.001	0.189
	Specialist 2	50.3 ± 6.3	49			
	Specialist 3	51.8 ± 5.5	52			

ICC = Intraclass correlation coefficient. All ICCs were calculated using a two-way random-effects model for absolute agreement. An ICC > 0.75 indicates good reliability

^a^*P*^f^ (Friedman t test)

^b^*P*^ICC^ (Intraclass Correlation)

### Readability of AI-generated responses

The readability of the AI-generated responses, assessed across six standardized indices, demonstrated significant variations between platforms (Table 5). Shapiro-Wilk tests confirmed that all readability indices followed a normal distribution. Statistically significant differences were identified for three key metrics: Flesch Reading Ease (FRE) [F(3,56) = 5.19, *P*=.003, η²=0.22], Gunning Fog Index [F(3,56) = 3.11, *P*=.034, η²=0.14], and Coleman-Liau Index (CLI) [F(3,56) = 9.22, *P*<.001, η²=0.33].

**Table 5:** Readability metrics across AI platforms (Mean ± SD)

_Metrics_	Mean ± sd				One-way-ANOVA		Partial (η2)	Shapiro-Wilk	
	Claude3-Opus	Gemini2.0	ChatGPT-4o	DeepSeek	F	*P*-value		W	*P*-value
FRE	31.60 ± 11.18	33.25 ± 8.29	34.25 ± 8.80	21.84 ± 10.24	5.191	0.003**	0.218	0.969	0.129
FKGL	14.91 ± 2.99	13.24 ± 1.45	13.91 ± 2.15	14.52 ± 2.79	1.355	0.266	0.068	0.979	0.407
Gunning FOG	18.89 ± 4.46	15.57 ± 1.67	16.27 ± 1.97	16.88 ± 3.58	3.107	0.034*	0.143	0.904	0.208
SMOG	13.50 ± 2.12	12.52 ± 1.36	12.71 ± 1.48	12.79 ± 2.77	0.674	0.571	0.035	0.987	0.758
CLI	15.25 ± 1.67	15.95 ± 1.52	15.14 ± 1.74	18.03 ± 1.88	9.223	< 0.001**	0.331	0.977	0.332
ARI	15.98 ± 3.61	13.83 ± 1.60	14.44 ± 2.13	14.48 ± 3.53	1.543	0.213	0.076	0.971	0.165

Abbreviations: FRE, Flesch Reading Ease; FKGL, Flesch-Kincaid Grade Level; SMOG, Simple Measure of Gobbledygook; CLI, Coleman-Liau Index; ARI, Automated Readability Index

**Table 6:** Characteristics of Flesch Kincaid reading Ease(FRE) generated by different AI tools

AI tools(FRE)		Mean Difference (I-J)	*P*-value	Cohen’s d	Effect Size Interpretation	95% Confidence Interval	
						Lower Bound	Upper Bound
Claude3-Opus	Gemini 2.0	-1.65	0.999	0.170	Small	-11.3327	8.0327
	ChatGPT-4o	-2.65	0.999	0.273	Small	-12.3327	7.0327
	DeepSeek	9.76200*	0.047*	1.007	Large	0.0793	19.4447
Gemini 2.0	Claude3-Opus	1.65	0.999	0.170	Small	-8.0327	11.3327
	ChatGPT-4o	-1	0.999	0.103	Small	-10.6827	8.6827
	DeepSeek	11.41200*	0.013*	1.177	Large	1.7293	21.0947
ChatGPT-4o	Claude3-Opus	2.65	0.999	0.273	Small	-7.0327	12.3327
	Gemini 2.0	1	0.999	0.103	Small	-8.6827	10.6827
	DeepSeek	12.41200*	0.005*	1.280	Large	2.7293	22.0947
DeepSeek	Claude3-Opus	-9.76200*	0.047*	1.007	Large	-19.4447	-0.0793
	Gemini 2.0	-11.41200*	0.013*	1.177	Large	-21.0947	-1.7293
	ChatGPT-4o	-12.41200*	0.005*	1.280	Large	-22.0947	-2.7293

^a^*P*<.05 are considered statistically significant

Cohen’s d > 0.8 can be interpreted as large effect

None of the AI platforms met established readability guidelines for patient materials. The mean Flesch-Kincaid Grade Level ranged from 13.2 to 14.9, and Flesch Reading Ease scores ranged from 21.8 to 34.3, categorizing all outputs as “difficult” and corresponding to a college-level reading requirement.

Further post-hoc pairwise comparisons with Bonferroni correction clarified these patterns. For the primary readability measure FRE (Table 6), ChatGPT-4o generated significantly more readable text than DeepSeek (mean difference = 12.41, *P*=.005, Cohen’s d = 1.28, large effect). Similarly, DeepSeek’s responses were significantly less readable than those from all other platforms (all *P* <.05, Cohen’s d > 1.0). No significant differences were observed among Claude3-Opus, Gemini 2.0, and ChatGPT-4o after correction (all *P* >.05). A similar pattern was observed for CLI, where DeepSeek’s text complexity was significantly higher than all other models (all *P* ≤.009). (Supplementary Table 2S2)

### Stratified analysis of question types

To further clarify the performance of LLMs across targeted user group, stratified analyses were conducted for caregiver/patient-focused (*n* = 6) and clinician-focused (*n* = 9) questions (Supplementary Table 2S3). A clear pattern emerged three models (Claude 3 Opus, Gemini 2.0, and ChatGPT-4o). These models generated responses to caregiver/patient-focused questions that were significantly more readable than their responses to clinician-focused queries. ChatGPT-4o produced significantly more readable text for caregivers (FRE: 39.78 ± 6.82) compared to clinicians (FRE: 29.42 ± 7.60; mean difference = 10.36, t (13) = 2.76, *P* =.016). Similar, statistically significant differences in FRE were observed for Claude 3 Opus (mean diff. = 12.33, *P* =.027) and Gemini 2.0 (mean diff. = 10.57, *P* =.008). DeepSeek showed no significant adjustment in readability based on the target audience. All DISCERN score comparisons between caregiver- and clinician-focused responses were non-significant (all *P* >.39).

## Discussion

### Principal findings and interpretation

This study evaluated LLMs across the continuum of pediatric asthma communication by employing a question set that reflects needs of both clinicians and patients/caregivers. Our findings reveal that while these models achieve factual reliability on standardized queries, they exhibit significant and clinically relevant disparities in linguistic accessibility.

In this assessment, ChatGPT-4o achieved the highest mean score (51.87 ± 7.65), with over 90% of its responses rated above the median level, indicating its reliable performance in conveying pediatric asthma information. However, statistical analysis confirmed that all four platforms performed equivalently, as measured by the DISCERN instrument. This suggests that LLMs have attained a convergence in informational reliability, supporting their potential as adjunctive tools for clinician reference. [21–22].

Despite generally reliable performance, instances of incomplete or misleading information were noted even among responses categorized as “Good” (DISCERN score 51–62) by the instrument. For example, in Question 10 (post-exacerbation follow-up), ChatGPT-4o failed to specify the guideline-recommended “2–4 week” window, despite receiving a high DISCERN score. This discrepancy highlights a key limitation of the DISCERN instrument: it prioritizes overall information structure, reliability, and scope over granular clinical detail. Thus, a “Good” DISCERN rating does not guarantee the precision of context-specific details critical for pediatric asthma management, reinforcing the necessity of expert oversight in AI-assisted patient communication.

In stark contrast to the comparable information quality, we identified significant and disparities in textual readability. All models failed to meet the AMA/NIH-recommended 6th- to 8th-grade reading level for patient materials [23–24], with mean outputs corresponding to college-level complexity [25]. The mean FRE scores (21.8–34.3) further classify the outputs as“difficult.”

The approximately 12.41-point difference between ChatGPT-4o and DeepSeek (*P* =.005, Cohen’s d = 1.28) is educationally instructive, representing a shift of more than one full U.S. grade level in comprehension demand [26]. Besides, Significant differences in the Gunning Fog Index may arise from variations in sentence complexity and dense terminology, which can impede reading flow. The significantly higher CLI for DeepSeek indicating that a greater use of complex, longer words, increases cognitive load [27]. Collectively, these disparities indicate tangible variations in the potential comprehensibility of AI-generated patient instructions. Our stratified analysis further reveals that three models (Claude 3 Opus, Gemini 2.0, and ChatGPT-4o) demonstrates a degree of contextual adaptation, generating responses to caregiver/patient-focused questions that were significantly more readable than their outputs for clinicians, as evidenced by higher FRE scores. DeepSeek showed no such capability, producing text of uniformly high complexity regardless of the audience. This finding clarifies that the models’ utility is context-dependent. while AI tools may serve as reference tools for clinicians, their suitability for direct patient communication varies considerably based on their capacity for linguistic simplification.

### Clinical implications and practical recommendations

The expanding role of AI technology within healthcare holds promise for both clinical decision support and patient care and education. The divergence between accuracy and accessibility necessitates a bifurcated implementation framework. For clinician-assistive applications, the priority remains factual accuracy and integration with authoritative medical databases. LLMs can function as efficient sources for preliminary information that requires expert verification.

For patient-facing or educational applications, readability must be elevated to a core performance metric. Poorly understandable instructions can precipitate adverse events [28]. Our data suggest that in the absence of simplification features, platforms like ChatGPT-4o, with their relatively higher baseline readability, are preferable for generating draft patient materials. Crucially, no current model is suitable for direct, unsupervised patient use.

A threshold informed by national surveys indicating median adult literacy was at the 6th-grade level [29]. This standard is further reinforced in pediatric care by CDC recommendations to optimize asthma education for caregivers with limited health literacy [30]. To bridge the accessibility gap, future development should integrate adaptive text simplification. This could involve simplifying sentence structures to organize content in a more understandable and logical manner, employing clear and plain language, providing comprehensive explanations [31] (e.g., footnotes), integrating visual elements and collecting educational background information to customize more targeted outputs.

Ultimately, LLMs should be positioned as adjuncts for professional judgment and humanized communication, with their application rigorously matched to the specific clinical context and patients’needs.

### Limitations and methodological considerations

Several methodological aspects must be considered when interpreting our findings.

Firstly, our 15 questions were derived from the GINA 2024 guideline, focusing on clear, evidence-based answers without including clinically controversial scenarios. This may overestimate LLM performance in real-world settings, where questions often lack definitive answers. Additionally, the limited sample size may be insufficient to detect subtle but clinically meaningful differences in DISCERN scores (e.g., between ChatGPT-4o and Gemini 2.0). Secondly, we did not assess the actual comprehension of AI-generated content by caregivers with varying health literacy levels, relying solely on objective readability indices. Third, our evaluation relied on objective readability indices and expert scoring, and it did not assess the actual comprehension of AI outputs by caregivers with varying health literacy levels, a critical area for future validation research. Additionally, the rapidly evolving nature of AI models posed challenges for evaluation. This study focused on the current versions of AI models and our findings may not apply to future iterations, necessitating further reassessments.

## Conclusion

Large Language Models (LLMs) demonstrate a dual potential in pediatric asthma care: they are clinically reliable but linguistically inaccessible. As decision-support tools for clinicians, these platforms can facilitate clinical reference. However, for direct patient education and communication, current LLMs are not yet fit for purpose due to a critical and uniform deficit in readability. Therefore, Future development advance on two parallel tracks: enhancing the clinical reasoning and verification capabilities of LLMs for professional support, while fundamentally prioritizing readability-by-design for public-oriented communication. Healthcare providers should engage with LLMs, rigorously safeguarding the clarity and safety of information delivered to patients.

## Supplementary Information

Below is the link to the electronic supplementary material.

Supplementary Material 1

Supplementary Material 2

Supplementary Material 3

## Data Availability

Data is provided within the manuscript or supplementary information files.

## Abbreviations

NLP: Natural Language Processing
AI: Artificial Intelligence
LLM: Large Language Models
ML: Machine Learning
FRE: Flesch Reading Ease
FKGL: Flesch-Kincaid Grade Level
SMOG: Simple Measure of Gobbledygook
CLI: Coleman-Liau Index
ARI: Automated Readability Index
ICC: Intraclass correlation coefficient
