# Supplementary material for: Evaluation of large Language models on pediatric asthma: a comparative study of Claude3-Opus, Gemini 2.0, ChatGPT-4o, and DeepSeek—a cross-sectional questionnaire study
Source: BMC Med Inform Decis Mak. 2026 Feb 10;26:77. doi: 10.1186/s12911-026-03371-x (PMC12990414; doi:10.1186/s12911-026-03371-x)
Supplement: Supplementary file 1 — Supplementary Material 1 [file 12911_2026_3371_MOESM1_ESM.docx]

**Supplementary Table 1.** The scoring criteria for the DISCERN instrument.

| **Questions (Total=16)** | **Points** |
| --- | --- |
| **Section1. Is the publication reliable?** |  |
| Question 1. Are the aims clear? | 1-5 |
| Question 2. Does it achieve its aims? | 1-5 |
| Question 3. Is it relevant? | 1-5 |
| Question 4. Is it clear what sources of information were used to compile the publication (other than the author or producer)? | 1-5 |
| Question 5. Is it clear when the information used or reported in the publication was produced? | 1-5 |
| Question 6. Is it balanced and unbiased? | 1-5 |
| Question 7. Does it provide details of additional sources of support and information? | 1-5 |
| Question 8. Does it refer to areas of uncertainty? | 1-5 |
| **Section2. How good is the quality of information on treatment choices?** |  |
| Question 9. Does it describe how each treatment works? | 1-5 |
| Question 10. Does it describe the benefits of each treatment? | 1-5 |
| Question 11. Does it describe the risks of each treatment? | 1-5 |
| Question 12. Does it describe what would happen if no treatment is used? | 1-5 |
| Question 13. Does it describe how the treatment choices affect overall quality of life? | 1-5 |
| Question 14. Is it clear that there may be more than one possible treatment choice? | 1-5 |
| Question 15. Does it provide support for shared decision-making? | 1-5 |
| **Section3. Overall Rating of the Publication** |  |
| Question 16. Based on the answers to all of the above questions, rate the overall quality of the publication as a source of information about treatment choices. | 1-5 |

*1=No; 3=Partially; 5=Yes.
