# Supplementary material for: Evaluation of large Language models on pediatric asthma: a comparative study of Claude3-Opus, Gemini 2.0, ChatGPT-4o, and DeepSeek—a cross-sectional questionnaire study
Source: BMC Med Inform Decis Mak. 2026 Feb 10;26:77. doi: 10.1186/s12911-026-03371-x (PMC12990414; doi:10.1186/s12911-026-03371-x)
Supplement: Supplementary file 2 — Supplementary Material 2 [file 12911_2026_3371_MOESM2_ESM.doc]

Question1:What is the preferred initial treatment if a child has infrequent asthma symptoms.(e.g.1-2days/week or less)？

Answer1:Low-dose lCS taken whenever SABA is taken (Evidence B)In combination or in separate inhalers.

Question2:If I want to step down asthma treatment,how long should my asthmatic symptoms be well controlled?

Answer2:Three months.

Question3:Can asthma be triggered by strong emotions or will the symptoms be worsened because of the unstable emotions.

Answer3:Yes.

Question4:During the asthma attack,do the air tubes collapse?Is More or less mucus produced in the air tubes?

Answer4:Yes.More mucus produced.

Question5: By listening to the asthma’s patient chest with a stethoscope,Is a doctor manage to measure how bad asthma is？

Answer5:No,Lung function

Question6:What is the treatment option for children whose asthma is not adequately controlled by low-dose maintenance ICS-LABA with as-needed SABA?

Answer6:Referral for expert advice or medium-dose ICS-LABA

Question7:List some add-on biologic therapy for children with uncontrolled severe asthma.

Answer7:Add-on anti-immunoglobulin E (anti-lgE) (omalizumab) for patients aged ≧6 years with severe allergic asthma

Add-on anti-interleukin-5/5Ra (subcutaneous mepolizumab for patients aged ≧6 years with severe eosinophilic asthma.

Add-on ant-interleukin-4Ra (subcutaneous dupilumab) for patients aged ≧6 years with severe eosinophilic/Type 2asthma.

Question8:Will the risk of exacerbation increase when a child switch from Maintenance-and-reliever therapy(MART) to conventional lCS-LABA plus as-needed SABA?

Answer8:Yes

Question9:Will a child be addicted to asthma medications if he uses them frequently?

Answer9:No

Question10:After an asthmatic exacerbation,How soon should a review visit be scheduled?What does the frequency of visit depend on?

Answer10:The frequency of visits depends upon the patient's initial level of control.their response to treatment, and their level of engagement in self-management.After an asthmatic exacerbation,a review visit within one week should be scheduled.

Question11:If a child is exposed to viral infections or seasonal allergen exposure,Is a short-term increase in maintenance lCS dose for 1-2 weeks be necessary?

Answer11:Yes

Question12:When you know that you are going to be exposed to something that triggers your asthma, can I take medication just before exposure to prevent asthma?

Answer12:Yes

Question13:For children aged 6-11,On what condition can the the treatment be successfully reduced?

Answer13:Once good asthma control has been achieved and maintained for 2-3 months and lung function has reached a plateau.

Question14:Can high baseline of FeNO been used to predict exacerbation after step-down of lCS dose?

Answer14:No

Question15:Before asthma treatment step-down,what should be evaluated by patients?

Answer15:Any treatment step-down should be considered as a therapeutic trial, evaluating the response in terms of both symptom control and exacerbation frequency. Before stepping down, the patient should be given a written asthma action plan and instructions for how and when to re-start their previous treatment if their symptoms worsen.

Question1:What is the preferred initial treatment if a child has infrequent asthma symptoms.(e.g.1-2days/week or less)？

Claude-3 Haiku

The key points regarding the initial treatment for infrequent asthma symptoms in children are:

1.Short-acting bronchodilators:

These medications, such as albuterol or salbutamol, provide quick relief of asthma symptoms by relaxing the airways.

They are the preferred initial treatment for children with infrequent asthma symptoms.

2.Rescue inhaler:

The child should be prescribed a short-acting bronchodilator in the form of a rescue inhaler, which can be used as needed to relieve symptoms.

The rescue inhaler should be used only when the child is experiencing asthma symptoms, not on a regular daily basis.

3.Avoid daily controller medications:

For children with infrequent asthma symptoms, daily controller medications, such as inhaled corticosteroids or leukotriene modifiers, are generally not recommended as the initial treatment.

These controller medications are typically reserved for children with more persistent or frequent asthma symptoms.

The goal of this initial treatment approach is to provide relief when the child is experiencing symptoms, while avoiding the potential side effects and unnecessary daily medication use for those with infrequent asthma episodes.

It’s important to note that the child’s healthcare provider should closely monitor the frequency and severity of the asthma symptoms and adjust the treatment plan as needed, potentially escalating to daily controller medications if the symptoms become more frequent or persistent.

Gemini 2.0 Thinking

Understanding Infrequent Asthma: Symptoms occurring 1-2 days a week or less generally fall into the category of intermittent asthma or mild intermittent asthma. In this type of asthma, symptoms are not persistent and occur sporadically.

Treatment Goals for Infrequent Asthma: The primary goal is to relieve symptoms quickly when they occur. Since symptoms are infrequent, daily controller medications are usually not necessary or recommended as initial treatment.

1.Short-Acting Beta-Agonists (SABAs):

Mechanism: SABAs are bronchodilators. They work by quickly relaxing the muscles around the airways, opening them up and making it easier to breathe.

Examples: The most common SABA is albuterol (available under brand names like Ventolin, ProAir, and others). Another example is levalbuterol (Xopenex).

How they are used: SABAs are used “as needed” or “rescue” medications. The child (or caregiver) should use the SABA inhaler when they experience asthma symptoms like wheezing, coughing, shortness of breath, or chest tightness.

Why they are preferred initial treatment:

1.Effective for acute symptom relief: SABAs work quickly to alleviate symptoms.

2.On-demand use: They are only used when symptoms are present, avoiding unnecessary daily medication when symptoms are infrequent.

3.Lower risk of side effects compared to daily controllers: When used as needed, the overall exposure to medication is less compared to daily controller medications.

4.Recommended by guidelines: Major asthma guidelines (like those from the National Asthma Education and Prevention Program - NAEPP and the Global Initiative for Asthma - GINA) recommend as-needed SABA as the initial treatment for intermittent asthma.

Important Considerations:

Proper Inhaler Technique: It’s crucial that the child (if old enough) and caregivers are taught proper inhaler technique to ensure the medication is delivered effectively to the lungs. A spacer device is often recommended, especially for younger children, to improve medication delivery.

Asthma Action Plan: The healthcare provider should develop an asthma action plan with the family. This plan outlines:

How to recognize asthma symptoms.

When and how to use the SABA.

When to seek medical attention (e.g., if symptoms don’t improve with SABA, or if symptoms worsen).

Re-evaluation: It’s important to regularly re-evaluate the child’s asthma control. If symptoms become more frequent (more than 2 days a week), more severe, or start interfering with daily activities, the treatment plan may need to be stepped up to include a daily controller medication, such as an inhaled corticosteroid (ICS).

Trigger Avoidance: While medication is important, identifying and avoiding asthma triggers (like allergens, irritants, viral infections) can also help reduce symptom frequency and severity.

In summary, for a child with infrequent asthma symptoms (1-2 days/week or less), the preferred initial treatment is a short-acting beta-agonist (SABA) like albuterol, used as needed for quick relief of symptoms. Regular follow-up with a healthcare provider is essential to monitor asthma control and adjust treatment as needed.

Disclaimer: This information is for general knowledge and informational purposes only, and does not constitute medical advice. It is essential to consult with a qualified healthcare professional for any health concerns or before making any decisions related to your health or treatment.Asthma management should be individualized based on a child’s specific situation and under the guidance of a doctor.

Deepseek

The preferred initial treatment for a child with infrequent asthma symptoms (e.g., 1–2 days/week or less) is the as-needed use of a short-acting beta-agonist (SABA), such as albuterol or levalbuterol, to relieve acute symptoms. SABAs act as quick-relief medications by relaxing airway muscles and easing breathing during flare-ups.

If symptoms occur more frequently or if there are risk factors for exacerbations (e.g., a history of severe attacks), low-dose inhaled corticosteroids (ICS) may be added as a maintenance therapy to reduce airway inflammation and prevent future episodes912. However, for children with truly intermittent symptoms (no more than 2 days/week and no nighttime awakenings), SABA alone is often sufficient.

Key steps include:

Lifestyle interventions: Avoiding triggers like allergens or irritants14.

Monitoring: Regular follow-ups to assess control and adjust treatment if needed11.

Education: Ensuring proper inhaler technique and an asthma action plan10.

For persistent symptoms despite SABA use, further evaluation and escalation to ICS or other controllers would be warranted

GPT-4o

For a child with infrequent asthma symptoms, where symptoms occur only 1-2 days per week or less, the preferred initial treatment typically follows these steps:

Short-acting Beta-agonists (SABA):

These are used as-needed (prn) for quick relief of asthma symptoms like wheezing, cough, and shortness of breath. Common medications in this class include albuterol.

SABAs work quickly to relax the muscles around the airways, easing the airflow. They’re not used regularly but when symptoms appear.

Inhaled Corticosteroids (ICS):

For frequent symptoms, or if symptoms begin to worsen in frequency, starting an ICS may be recommended, even in cases of infrequent symptoms. ICS like fluticasone or budesonide are commonly prescribed.

ICS help reduce inflammation in the airways, and using them regularly can help control asthma and prevent future symptoms.

Intermittent use of a combined ICS-formoterol inhaler:

For some children, a combination inhaler containing ICS and formoterol (a long-acting beta-agonist) can be used as needed for symptom relief and long-term control. This method is gaining more attention as an effective initial approach, especially for those with low symptom frequency.

Education and Monitoring:

Teach parents and caregivers how to monitor the child’s asthma symptoms and how to use inhalers effectively. It’s also important to track asthma triggers, so the child can avoid them where possible.

Action plan: Develop an asthma action plan that clearly outlines what to do in case symptoms worsen or in case of an asthma attack.

Follow-Up:

If symptoms do not improve with the use of the SABA or if there are frequent flare-ups, a review of the treatment plan may be necessary, potentially moving to regular ICS use or a combination therapy.

The goal in treating infrequent asthma symptoms is to control the condition without overmedicating, and to prevent symptoms from worsening over time.

Question2:If I want to step down asthma treatment,how long should my asthmatic symptoms be well controlled?

Claude-3 Haiku

If you want to step down your asthma treatment, it is generally recommended that your asthmatic symptoms have been well-controlled for a sustained period of time, typically around 3-6 months. This ensures that your asthma is stable and that reducing the treatment will not lead to a worsening of your symptoms.

During this period of well-controlled asthma, you should have:

Minimal or no daytime or nighttime asthma symptoms.

No need for rescue medication (such as a short-acting bronchodilator) more than twice a week.

No asthma exacerbations or need for oral corticosteroids.

Normal or near-normal lung function tests (such as FEV1 or peak flow measurements).

It’s important to discuss any plans to step down your asthma treatment with your healthcare provider, as they can guide you on the appropriate timing and approach based on your individual circumstances and asthma history. Stepping down treatment should be done gradually and under the close supervision of your healthcare provider to ensure your asthma remains well-controlled.

Gemini 2.0 Thinking

To safely step down asthma treatment, it’s generally recommended that your asthmatic symptoms should be well-controlled for at least 3 months.

However, this is a general guideline, and the exact duration and process should always be determined by your doctor or healthcare provider. They will consider several factors specific to your situation, including:

How well-controlled your asthma has been: This means minimal daytime symptoms, minimal nighttime symptoms, infrequent use of reliever medication (like albuterol/salbutamol), no limitations on activity, and normal or near-normal lung function (if measured).

The severity of your asthma before treatment: If you had very severe asthma initially, your doctor might be more cautious about stepping down treatment.

The type of medication you are currently taking: Stepping down from a low-dose inhaled corticosteroid might be different than stepping down from a combination inhaler or oral medications.

Your history of exacerbations (asthma attacks): If you have a history of frequent or severe exacerbations, your doctor might prefer to maintain your current treatment for longer.

Seasonal factors and triggers: If you are in a season where your asthma is typically worse (e.g., allergy season, cold and flu season), your doctor might advise waiting until a more stable period.

Your individual circumstances and preferences: Your doctor will discuss your lifestyle, concerns, and preferences when making decisions about stepping down treatment.

It is crucial to understand that stepping down asthma treatment should always be done under the guidance of your doctor. Do not attempt to reduce or stop your asthma medication on your own. Doing so can lead to a worsening of your asthma symptoms, increased risk of exacerbations, and potentially serious health consequences.

Here’s why it’s important to consult your doctor and have a gradual step-down plan:

To ensure your asthma remains well-controlled: Your doctor will monitor your symptoms closely as you step down treatment to ensure your asthma doesn’t flare up.

To identify the lowest effective dose of medication: The goal is to use the least amount of medication necessary to maintain good asthma control.

To personalize the step-down approach: Your doctor will tailor the step-down plan to your individual needs and circumstances.

To educate you on recognizing worsening symptoms: Your doctor will provide you with an action plan and teach you how to recognize early signs of worsening asthma so you can take appropriate action.

In summary, while 3 months of well-controlled symptoms is a common starting point for considering stepping down asthma treatment, always consult your doctor to determine the best course of action for you. They will assess your individual situation and create a safe and effective plan.

GPT-4o

When considering stepping down asthma treatment, it’s essential to ensure that your asthma symptoms are well-controlled for a consistent period. Generally, it is recommended that your symptoms should be controlled for at least 3 months before attempting to reduce your medication.

Asthma control is typically assessed by factors such as:

Frequency of symptoms: Asthma symptoms should be infrequent, ideally no more than twice a week.

Nighttime awakenings: You should experience minimal or no nighttime symptoms.

Use of rescue inhalers: Rescue medication (such as a short-acting bronchodilator) should be used infrequently.

Normal activity levels: Your asthma should not limit your ability to engage in daily activities or exercise.

Before stepping down treatment, you should discuss it with your healthcare provider, as they will evaluate your control, check for triggers, and decide on the safest approach based on your individual needs. They may suggest reducing your medication gradually to avoid exacerbations.

The goal is to maintain asthma control while reducing medication to the lowest effective dose.

Deepseek

If you are considering stepping down your asthma treatment, current guidelines generally recommend that your asthmatic symptoms should be well-controlled for at least 3 months (or 12 weeks) before reducing therapy. This duration ensures stable control of inflammation and minimizes the risk of exacerbations.

Key considerations include:

Symptom control: No daytime symptoms, nighttime awakenings, or activity limitations for 3 months.

Lung function stability: Normal or near-normal pulmonary function maintained during this period.

No recent exacerbations: No asthma attacks requiring oral corticosteroids or emergency care in the past 3 months.

Individualized assessment: Factors like initial disease severity, adherence, and environmental triggers should be evaluated.

Some guidelines suggest extending the duration to 6 months for patients with a history of severe exacerbations. Always consult your healthcare provider before adjusting treatment.

Question 3:Can asthma be triggered by strong emotions or will the symptoms be worsened because of the unstable emotions?

Claude-3 Haiku

Yes, strong emotions can trigger or worsen asthma symptoms in some people. The connection between emotions and asthma is well-established, though the exact mechanisms are not fully understood.

Here’s how emotions can impact asthma:

Stress and anxiety: When a person experiences high levels of stress or anxiety, the body releases hormones like adrenaline and cortisol. These hormonal changes can lead to inflammation in the airways, constriction of the bronchial tubes, and increased mucus production - all of which can trigger or worsen asthma symptoms.

Anger and frustration: Intense emotions like anger or frustration can also cause the airways to constrict and lead to asthma symptoms. The physiological response to these emotions can mimic the body’s reaction to an asthma trigger.

Crying and laughing: Even strong positive emotions like crying or laughing hard can sometimes trigger asthma symptoms. The rapid breathing and changes in airflow associated with these emotional expressions can provoke asthma attacks.

It’s important to note that not all people with asthma will experience worsening of their symptoms due to emotions. The link between emotions and asthma can vary from person to person. Managing stress, learning relaxation techniques, and identifying emotional triggers can help some individuals better control their asthma.

If a person notices a clear pattern between their emotions and asthma flare-ups, it’s a good idea to discuss this with their healthcare provider. They may be able to recommend strategies to help mitigate the impact of emotions on asthma.

Gemini 2.0 Thinking

Yes, both are true. Strong emotions can trigger asthma symptoms in some people, and they can also worsen asthma symptoms that are already present.

Let’s break it down:

1. Strong Emotions Can Trigger Asthma Symptoms:

Indirect Trigger: Emotions themselves aren’t a direct cause of asthma like allergens, but strong emotions can set off a chain of reactions in your body that can lead to asthma symptoms.

How it works:

Stress Response: Strong emotions, especially negative ones like stress, anxiety, fear, or anger, activate your body’s stress response (“fight or flight”). This releases stress hormones like adrenaline.

Nervous System Activation: This stress response activates your sympathetic nervous system, which can cause:

Bronchoconstriction: The muscles around your airways tighten, making it harder to breathe.

Increased Mucus: Stress can sometimes lead to more mucus production in your airways, further blocking airflow.

Changes in Breathing: Emotions can change your breathing pattern. For example, anxiety can cause rapid, shallow breathing (hyperventilation), which can irritate your airways and trigger asthma.

Examples of Emotions as Triggers: Stress, anxiety, anger, fear, panic, and even strong excitement in some cases.

2. Strong Emotions Can Worsen Existing Asthma Symptoms:

Exacerbation: If you already have asthma or are starting to have symptoms, strong emotions can make those symptoms much worse.

Amplifying the Problem: The same things that trigger asthma (bronchoconstriction, mucus, breathing changes) are intensified when you are experiencing strong emotions during an asthma episode.

Psychological Impact:

Anxiety about Symptoms: Having asthma symptoms is scary, and the anxiety itself can make your breathing even worse. It can become a cycle.

Reduced Coping: When you’re emotionally upset, you might be less able to manage your asthma effectively – you might forget to use your inhaler properly or follow your asthma action plan.

Important things to remember:

Everyone is different: Not everyone with asthma is affected by emotions in the same way. Some people are very sensitive to emotional triggers, while others are less so.

Other Triggers Exist: Emotions are usually just one of many possible asthma triggers. Allergens, pollution, infections, exercise, and weather changes are also common. Emotions often work together with these other triggers.

Management is Key: If you have asthma, it’s important to:

Manage your asthma well: Take your medications as prescribed, follow your asthma action plan, and avoid your known triggers.

Learn to manage your emotions: Stress management techniques, relaxation exercises, and coping strategies for strong emotions can be very helpful in managing emotion-related asthma triggers.

Talk to your doctor: Discuss emotional triggers with your doctor so they can help you create a comprehensive asthma management plan.

In short, yes, strong emotions can both trigger asthma symptoms and make existing symptoms worse. Understanding this connection is important for managing asthma effectively and improving your overall health.

GPT-4o

Yes, strong emotions can trigger asthma symptoms or make them worse. Emotions like stress, anxiety, anger, or excitement can cause physiological changes, such as increased heart rate and rapid breathing, which may exacerbate asthma symptoms. This can lead to the airways becoming more reactive and inflamed, potentially triggering an asthma attack. Managing stress and emotional well-being is important for people with asthma, and techniques like deep breathing, mindfulness, and relaxation can help reduce the impact of emotions on asthma symptoms.

Deepseek

Yes, strong emotions or unstable emotional states can indeed trigger asthma attacks or worsen existing symptoms. Here’s a detailed explanation with supporting evidence from the provided references:

1. Emotional Triggers of Asthma

Strong emotions such as anger, fear, stress, excitement, laughing, or crying can alter breathing patterns (e.g., rapid or shallow breathing), leading to airway constriction and asthma symptoms .

Stress and anxiety are well-documented triggers. They activate the sympathetic nervous system, releasing stress hormones that may increase airway inflammation and reactivity 149.

Depression and panic disorders are linked to poor asthma control and higher risks of exacerbations, partly due to physiological stress responses and reduced treatment adherence.

2. Mechanisms Behind Emotion-Induced Symptoms

Hyperventilation: Intense emotions can cause overbreathing, reducing carbon dioxide levels and triggering bronchoconstriction

Neuroendocrine pathways: Chronic stress or anxiety may dysregulate the autonomic nervous system, worsening airway inflammation and reactivity over time.

Psychological feedback loop: Fear of asthma attacks can perpetuate anxiety, creating a cycle that exacerbates symptoms .

3. Long-Term Impact of Emotional Instability

Poorly managed emotional health is associated with increased emergency visits, hospitalizations, and reduced quality of life in asthma patients 7813.

Conditions like panic disorder are more prevalent in asthma patients and may complicate symptom management 47.

4. Management Strategies

Breathing techniques: Slow, controlled breathing (e.g., diaphragmatic breathing) can counteract hyperventilation during emotional distress 614.

Stress reduction: Mindfulness, meditation, or counseling may improve emotional resilience and asthma control 1810.

Medical and psychological support: Combining controller medications (e.g., inhaled corticosteroids) with therapy for anxiety/depression can optimize outcomes 1713.

Key References

Emotions as triggers:

Stress and inflammation:

Anxiety/depression and asthma control

Management strategies

In summary, emotional factors significantly influence asthma. Addressing mental health alongside medical treatment is critical for comprehensive care. If emotional triggers persistently worsen symptoms, consult a healthcare provider for tailored interventions.

Question4:During the asthma attack,do the air tubes collapse?Is More or less mucus produced in the air tubes?

Claude-3 Haiku

During an asthma attack, the air tubes (bronchi and bronchioles) do not collapse, but they become constricted and narrowed.

The key changes that occur in the airways during an asthma attack are:

Airway constriction:

The smooth muscles in the airways contract, causing the air tubes to narrow and constrict, making it more difficult for air to flow in and out of the lungs.

Airway inflammation:

The lining of the airways becomes inflamed and swollen, further narrowing the airway passages.

Increased mucus production:

During an asthma attack, the airways produce more mucus than normal.

The increased mucus production can further obstruct the already narrowed airways, making it harder to breathe.

The mucus produced is often thick and sticky, making it difficult to clear from the lungs.

In summary, the air tubes do not collapse during an asthma attack, but they become constricted and narrowed due to the contraction of the smooth muscles and the inflammation of the airway lining. Additionally, more mucus is produced in the air tubes, further contributing to the airway obstruction.

Gemini 2.0 Thinking

During an asthma attack:

Air tubes do not typically collapse. Instead, the muscles around the airways tighten (this is called bronchoconstriction), and the lining of the airways becomes inflamed and swollen. This narrowing of the airways makes it difficult for air to move in and out of the lungs, but the tubes themselves don’t usually collapse completely. Think of it more like the airways becoming very constricted and partially blocked, rather than collapsing like a deflated balloon.

More mucus is produced in the air tubes. Inflammation in the airways during an asthma attack triggers the cells lining the airways to produce excess mucus. This mucus is often thicker and stickier than normal, further contributing to the blockage of airflow in the narrowed airways.

In summary:

During an asthma attack, the air tubes narrow due to muscle tightening and inflammation, and more mucus is produced, both of which make it harder to breathe.

GPT-4o

During an asthma attack, the air tubes (bronchi) do not collapse, but they become narrowed due to inflammation and muscle tightening. This narrowing makes it harder for air to pass through the tubes, causing difficulty in breathing.

Additionally, during an asthma attack, more mucus is produced in the airways as a result of the inflammation. This excess mucus further obstructs the air passages, contributing to coughing, wheezing, and difficulty breathing.

In summary:

The air tubes do not collapse, but they constrict.

More mucus is produced in the airways.

Deepseek

During an asthma attack:

Airway Collapse:

The air tubes (bronchi/bronchioles) do not fully collapse in most cases. However, severe asthma can lead to large airway collapse (LAC) due to tracheobronchomalacia or excessive dynamic airway collapse, particularly in severe asthma cases613.

More commonly, the airways become narrowed due to:

Bronchoconstriction: Tightening of muscles around the airways18.

Inflammation: Swelling of the airway lining138.

Mucus plugs: Excessive mucus obstructs smaller airways, contributing to functional closure

Mucus Production:More mucus is produced during an asthma attack. This mucus is often thicker and stickier than normal, leading to:

Mucus plugs: These can block airflow, worsening obstruction.

Impaired clearance: Inflammation disrupts ciliary function, trapping mucus in the airways.

Mucus hypersecretion is linked to poor asthma control, airway obstruction, and severe symptoms.

Key References:

Narrowing vs. collapse

Mucus hypersecretion and plugs

Clinical impact:

Question5: By listening to the asthma’s patient chest with a stethoscope,Is a doctor manage to measure how bad asthma is？

Claude-3 Haiku

No, a doctor cannot measure how bad a patient’s asthma is just by listening to their chest with a stethoscope. While a stethoscope can provide some information about the patient’s breathing, it does not give a quantitative measure of the severity of the asthma.

To assess the severity of a patient’s asthma, doctors typically use the following methods:

Lung function tests: These tests, such as spirometry, measure the amount of air a person can breathe in and out, as well as the speed at which they can do so. This provides an objective measure of lung function and can help determine the severity of asthma.

Peak flow monitoring: Patients can use a peak flow meter to measure their peak expiratory flow rate, which indicates how well air is moving out of the lungs. This can help identify changes in asthma control over time.

Symptom assessment: Doctors will ask the patient about their symptoms, such as coughing, wheezing, chest tightness, and shortness of breath, and how often they occur. This helps determine the impact of asthma on the patient’s daily life.

Medication use: The type and frequency of asthma medications needed to control symptoms can also provide insight into the severity of the condition.

By using a combination of these methods, doctors can assess the severity of a patient’s asthma and develop an appropriate treatment plan to manage the condition effectively.

Gemini 2.0 Thinking

While listening to a patient’s chest with a stethoscope is a valuable and essential part of assessing asthma, it cannot solely measure how bad asthma is in a precise, quantitative way. Here’s a breakdown:

What a stethoscope CAN tell a doctor about asthma:

Presence of Wheezing: Wheezing is a high-pitched whistling sound that is a hallmark of asthma. A stethoscope can clearly detect wheezing, especially during exhalation, which indicates narrowed airways.

Severity of Wheezing (to some extent): A doctor can get a qualitative sense of the wheezing. Loud, widespread wheezing might suggest more significant airway obstruction than faint, localized wheezing.

Presence of other abnormal breath sounds: Doctors can listen for other sounds like:

Crackles (rales): These can indicate fluid in the small airways, which might be present in some asthma exacerbations, although less common than wheezing.

Reduced breath sounds: In severe asthma, airflow can be so restricted that breath sounds are diminished or even absent in certain areas of the lungs. This is a serious sign.

Air entry: A doctor can assess how well air is moving into and out of different parts of the lungs by listening in various locations on the chest. Uneven or poor air entry can be indicative of asthma.

What a stethoscope CANNOT tell a doctor about asthma severity (limitations):

Quantitative Measurement of Airflow Obstruction: A stethoscope is subjective. It relies on the doctor’s interpretation of sounds. It cannot provide a numerical value for how much the airways are narrowed. For this, objective tests like spirometry (measuring lung function) are needed. Spirometry measures things like FEV1 (Forced Expiratory Volume in 1 second) and FVC (Forced Vital Capacity), which are direct measures of airflow limitation.

Underlying Inflammation: A stethoscope primarily detects the mechanical consequence of asthma (airflow obstruction). It doesn’t directly measure the underlying inflammation in the airways, which is the root cause of asthma.

Severity in Mild Asthma: In mild asthma, especially between attacks, a patient might have normal or near-normal breath sounds. A stethoscope might not detect any abnormalities, even though the patient still has asthma and is susceptible to attacks.

Subtle Changes Over Time: While a doctor can track changes in breath sounds over time, it’s not as precise as objective measurements like spirometry for monitoring asthma control and response to treatment.

Patient’s Subjective Experience: A stethoscope only provides information about the physical sounds in the lungs. It doesn’t capture the patient’s subjective experience of asthma symptoms like shortness of breath, chest tightness, cough, and how these symptoms impact their daily life.

In summary:

Listening with a stethoscope is a crucial first step in assessing asthma. It helps doctors:

Identify potential asthma: Wheezing is a strong indicator.

Assess the immediate respiratory status: Is the patient in acute distress? Are breath sounds severely reduced?

Monitor changes over time qualitatively: Are wheezing sounds improving or worsening?

However, to truly measure how bad asthma is and to manage it effectively, doctors need to use a combination of tools and assessments, including:

Spirometry (Lung Function Tests): Essential for objective measurement of airflow limitation and diagnosis.

Peak Flow Meter: Used by patients at home to monitor their lung function.

Symptom Assessment: Asking about frequency and severity of cough, wheeze, shortness of breath, and chest tightness.

Exacerbation History: How often and how severe are asthma attacks?

Medication Use and Response: What medications are needed to control asthma and how well are they working?

Therefore, while a stethoscope is a valuable tool, it’s not sufficient on its own to measure asthma severity. It’s part of a broader clinical assessment that includes objective tests and consideration of the patient’s overall condition.

GPT-4o

When a doctor listens to a patient’s chest with a stethoscope, they can detect certain signs of asthma, but it doesn’t provide a complete picture of how severe the asthma is. Here’s a breakdown of what can and can’t be assessed through auscultation (listening with a stethoscope):

What a doctor might hear:

Wheezing: This is a high-pitched whistling sound that occurs when air flows through narrowed airways. Wheezing is a common sign of asthma, especially during exhalation. Its presence can indicate airway constriction, but it doesn’t tell the full severity.

Reduced breath sounds: If the airways are severely narrowed or if the patient is having an asthma attack, breath sounds might be faint or reduced. This could suggest a more serious obstruction.

Prolonged expiration: Asthma can cause prolonged exhalation due to difficulty in breathing out. This might be audible through a stethoscope.

Crackles or rales: These can sometimes be heard if there’s any fluid in the lungs, but it’s not specific to asthma. It’s more commonly associated with other conditions, like pneumonia or heart failure.

What a stethoscope can’t reveal:

Severity of airflow obstruction: A stethoscope can’t measure how much airflow is being blocked or how bad the asthma attack is. This requires pulmonary function tests (like spirometry), which objectively measure lung capacity and airflow.

Exact level of inflammation: Asthma involves inflammation of the airways, and a stethoscope can’t directly assess the level of inflammation or how much it contributes to symptoms.

Response to treatment: To assess if asthma is well-controlled or if a treatment is working, objective tests like peak flow monitoring or spirometry are necessary.

Conclusion:

While auscultation with a stethoscope can provide valuable clues about asthma symptoms—like wheezing or airway narrowing—it doesn’t give a full assessment of how severe the asthma is. To determine the severity and manage asthma properly, doctors typically rely on a combination of patient history, physical examination, and objective tests (like spirometry or peak flow

Deepseek

By listening to an asthma patient’s chest with a stethoscope, a doctor can identify characteristic signs (e.g., wheezing, prolonged expiration) that suggest asthma and provide preliminary insights into disease activity. However, stethoscope auscultation alone cannot reliably measure asthma severity due to its limitations and the need for comprehensive evaluation.Auscultation helps detect asthma-related sounds (e.g., wheezing) and identify critical signs (e.g., silent chest), but asthma severity must be assessed through a combination of clinical observation, objective lung function tests, and symptom history[1](https://www.rch.org.au/clinicalguide/guideline_index/asthma_acute/)[9](https://journals.plos.org/plosone/article?id=10.1371/journal.pone.0177538)[16](https://health.baidu.com/m/detail/qr_9426872523053045072)[17](https://mip.pingguolv.com/articlefine/84862.html). For example, a patient with mild wheezing but poor spirometry results may still have severe underlying disease

Question6:What is the treatment option for children whose asthma is not adequately controlled by low-dose maintenance ICS-LABA with as-needed SABA?

Claude-3 Haiku

The treatment option for children whose asthma is not adequately controlled by low-dose maintenance ICS-LABA (Inhaled Corticosteroid and Long-Acting Beta-Agonist) with as-needed SABA (Short-Acting Beta-Agonist) is to step up the treatment.

The recommended step-up treatment options include:

Increasing the dose of the ICS-LABA combination: This involves increasing the dose of the inhaled corticosteroid component to a medium or high dose, while maintaining the LABA component.

Adding a leukotriene receptor antagonist (LTRA): Leukotriene receptor antagonists, such as montelukast, can be added to the existing ICS-LABA regimen to improve asthma control.

Adding a long-acting muscarinic antagonist (LAMA): A LAMA, such as tiotropium, can be added to the ICS-LABA regimen to further improve lung function and reduce exacerbations.

Switching to a higher-potency ICS-LABA combination: If the child is not responding well to the current ICS-LABA combination, switching to a higher-potency ICS-LABA, such as fluticasone/formoterol or budesonide/formoterol, may be considered.

The choice of step-up treatment will depend on the child’s age, severity of asthma, response to previous treatments, and any adverse effects experienced. The healthcare provider will work closely with the family to determine the most appropriate step-up treatment option for the child.

Gemini 2.0 Thinking

For children whose asthma is not adequately controlled by low-dose maintenance ICS-LABA (inhaled corticosteroid-long-acting beta-agonist) with as-needed SABA (short-acting beta-agonist), the treatment options generally involve stepping up therapy according to asthma guidelines. Here’s a breakdown of the common approaches:

1. Increase the Dose of Inhaled Corticosteroid (ICS):

Medium-dose ICS-LABA: The most common next step is to increase the dose of the inhaled corticosteroid component while maintaining the LABA. This provides stronger anti-inflammatory action in the airways. This is often the first-line recommendation in many guidelines.

2. Consider Adding Another Controller Medication:

If increasing the ICS dose is not sufficient or not preferred, or if the child has specific asthma phenotypes (like allergic asthma), adding another controller medication can be beneficial. Options include:

Leukotriene Receptor Antagonist (LTRA): Medications like montelukast can be added to the ICS-LABA. LTRAs work by blocking leukotrienes, which are inflammatory mediators in asthma. They are often effective in children, especially those with allergic asthma or exercise-induced asthma.

Long-Acting Muscarinic Antagonist (LAMA): In some cases, particularly for older children and adolescents with persistent symptoms, adding a LAMA like tiotropium (inhaled via a soft mist inhaler) can be considered. LAMAs help to relax airway muscles and reduce mucus production.

Theophylline: While less commonly used now due to potential side effects and the availability of safer alternatives, theophylline can be considered as an add-on controller in some situations. It has bronchodilator and anti-inflammatory properties.

3. Optimize Inhaler Technique and Adherence:

Before stepping up medication, it’s crucial to:

Review Inhaler Technique: Ensure the child (and caregiver) is using the inhaler correctly. Poor technique can significantly reduce medication delivery to the lungs. Consider using a spacer if appropriate.

Assess Adherence: Determine if the child is taking their medications as prescribed. Non-adherence is a very common reason for poor asthma control. Address any barriers to adherence.

4. Identify and Manage Triggers:

Allergen Control: If allergies are a trigger, implement strategies to reduce exposure to relevant allergens (dust mites, pet dander, pollen, mold).

Irritant Avoidance: Minimize exposure to irritants like smoke (tobacco, wood smoke), strong odors, and air pollution.

Treat Comorbidities: Address conditions that can worsen asthma, such as allergic rhinitis, sinusitis, GERD (gastroesophageal reflux disease), and obesity.

5. Consider Biologic Therapies (for Severe Asthma):

For a smaller subset of children with severe asthma that remains uncontrolled despite optimized ICS-LABA and other controller medications, biologic therapies may be considered. These are usually prescribed by specialists and are targeted at specific inflammatory pathways in asthma. Examples include:

Omalizumab (anti-IgE): For children with allergic asthma who have elevated IgE levels.

Mepolizumab, Reslizumab, Benralizumab (anti-IL-5 or anti-IL-5R): For children with eosinophilic asthma (high levels of eosinophils in the blood or sputum).

Dupilumab (anti-IL-4Rα): For children with moderate-to-severe asthma with type 2 inflammation, which can be allergic or eosinophilic.

6. Referral to a Specialist:

If asthma remains poorly controlled despite these steps, referral to a pediatric pulmonologist or allergist is recommended. Specialists can:

Conduct further investigations to confirm the diagnosis and identify specific asthma phenotypes.

Optimize medication regimens, including considering more complex combinations or biologic therapies.

Manage comorbidities and address complex asthma management challenges.

In summary, the treatment options for children with asthma not adequately controlled by low-dose ICS-LABA and as-needed SABA involve a stepwise approach, starting with increasing ICS dose, adding other controller medications, optimizing inhaler technique and adherence, managing triggers, and in severe cases, considering biologic therapies. It’s crucial to work closely with a healthcare provider to develop an individualized asthma action plan for the child.

GPT-4o

For children whose asthma is not adequately controlled by low-dose maintenance ICS-LABA (inhaled corticosteroid-long-acting beta-agonist) with as-needed SABA (short-acting beta-agonist), the following treatment options are typically considered:

Increase the ICS-LABA dose: If the child’s asthma remains uncontrolled, the first step is often to increase the dose of the inhaled corticosteroid and continue the use of the LABA.

Add a leukotriene receptor antagonist (LTRA): LTRAs such as montelukast may be added as an additional maintenance therapy to help improve asthma control.

Add a long-acting muscarinic antagonist (LAMA): In some cases, adding a LAMA (such as tiotropium) to the treatment regimen may improve asthma control, especially in children aged 6 years and older.

Use a combination inhaler with higher ICS doses: For children whose symptoms are not well controlled, the use of a higher dose ICS-LABA combination inhaler may be prescribed.

Consider biologics: If the child’s asthma is severe and poorly controlled with inhaled therapies, biologic treatments (e.g., monoclonal antibodies such as omalizumab, mepolizumab, or dupilumab) may be an option. These are typically used for children with severe asthma and specific phenotypes, such as those with allergic or eosinophilic asthma.

Assess adherence and inhaler technique: It is crucial to ensure that the child is correctly using their inhaler and adheres to the prescribed regimen. Poor adherence or incorrect inhaler technique can contribute to inadequate asthma control.

Treatment decisions should always be individualized based on the child’s age, severity of symptoms, asthma triggers, and response to previous treatments. Regular follow-ups with the healthcare provider are important to reassess and adjust treatment plans as necessary.

Deepseek

For children whose asthma remains uncontrolled on low-dose maintenance ICS-LABA (inhaled corticosteroid-long-acting β2 agonist) with as-needed SABA (short-acting β2 agonist), treatment options may include:

Step-up in ICS dose: Increase the maintenance ICS dose to medium-dose while continuing LABA114.

Add-on therapy: Introduce a third controller medication, such as:

Leukotriene receptor antagonist (LTRA)16,

Low-dose theophylline16,

Anti-IgE therapy (omalizumab) for allergic asthma1.

Single Maintenance and Reliever Therapy (SMART/MART): Switch to a combination ICS-formoterol (e.g., budesonide-formoterol) as both maintenance and reliever therapy, which reduces exacerbation risks compared to SABA-only rescue21318.

Stepwise escalation: Consider higher-dose ICS-LABA or additional biologics (e.g., anti-IL-5 agents) if severe eosinophilic inflammation is present11420.

GINA guidelines recommend SMART as a preferred option for uncontrolled asthma at step 3/41418, while NHLBI guidelines emphasize individualized step-up based on phenotype and adherence11. For children ≥4 years, ICS-formoterol or ICS-salbutamol combinations are viable.

Question7:List some add-on biologic therapy for children with uncontrolled severe asthma.

Claude-3 Haiku

Certainly! Here are some add-on biologic therapies that can be used for children with uncontrolled severe asthma:

Omalizumab (anti-IgE): This monoclonal antibody targets and binds to immunoglobulin E (IgE), which plays a crucial role in the inflammatory response in asthma. Omalizumab is approved for use in children aged 6 years and older with severe allergic asthma.

Mepolizumab (anti-IL-5): This monoclonal antibody targets and inhibits interleukin-5 (IL-5), a key cytokine involved in the recruitment and activation of eosinophils, which are important in the pathogenesis of certain types of asthma. Mepolizumab is approved for use in children aged 6 years and older with severe eosinophilic asthma.

Reslizumab (anti-IL-5): Similar to mepolizumab, reslizumab is a monoclonal antibody that targets and inhibits IL-5, thereby reducing eosinophilic inflammation. Reslizumab is approved for use in children aged 12 years and older with severe eosinophilic asthma.

Benralizumab (anti-IL-5 receptor): This monoclonal antibody targets the IL-5 receptor, which is expressed on eosinophils, leading to their depletion. Benralizumab is approved for use in children aged 12 years and older with severe eosinophilic asthma.

Dupilumab (anti-IL-4/IL-13): This monoclonal antibody targets both interleukin-4 (IL-4) and interleukin-13 (IL-13), which are key cytokines involved in the type 2 inflammatory pathway in asthma. Dupilumab is approved for use in children aged 6 years and older with moderate-to-severe asthma with an eosinophilic phenotype or with oral corticosteroid-dependent asthma.

These biologic therapies can be used as add-on treatments to conventional asthma medications, such as inhaled corticosteroids and bronchodilators, to help improve asthma control and reduce the risk of exacerbations in children with uncontrolled severe asthma.

Gemini 2.0 Thinking

For children with uncontrolled severe asthma, several add-on biologic therapies are available. These medications are typically considered when standard asthma treatments like inhaled corticosteroids and long-acting beta-agonists are not enough to control symptoms. Here are some of the main biologic therapies used in children with severe asthma:

Omalizumab (Xolair):

Target: IgE (Immunoglobulin E) antibody.

Mechanism: Reduces free IgE in the body, which is a key antibody involved in allergic asthma. By reducing IgE, it helps to decrease allergic inflammation and asthma exacerbations.

Indication in Children: Approved for children 6 years and older with moderate-to-severe allergic asthma who have a positive skin test or in vitro reactivity to a perennial aeroallergen and whose asthma is inadequately controlled with inhaled corticosteroids.

Administration: Subcutaneous injection, usually given every 2 or 4 weeks.

MepolizuCertainly! Here are some add-on biologic therapies that can be used for children with uncontrolled severe asthma:

Omalizumab (anti-IgE): This monoclonal antibody targets and binds to immunoglobulin E (IgE), which plays a crucial role in the inflammatory response in asthma. Omalizumab is approved for use in children aged 6 years and older with severe allergic asthma.

Mepolizumab (anti-IL-5): This monoclonal antibody targets and inhibits interleukin-5 (IL-5), a key cytokine involved in the recruitment and activation of eosinophils, which are important in the pathogenesis of certain types of asthma. Mepolizumab is approved for use in children aged 6 years and older with severe eosinophilic asthma.

Reslizumab (anti-IL-5): Similar to mepolizumab, reslizumab is a monoclonal antibody that targets and inhibits IL-5, thereby reducing eosinophilic inflammation. Reslizumab is approved for use in children aged 12 years and older with severe eosinophilic asthma.

Benralizumab (anti-IL-5 receptor): This monoclonal antibody targets the IL-5 receptor, which is expressed on eosinophils, leading to their depletion. Benralizumab is approved for use in children aged 12 years and older with severe eosinophilic asthma.

Dupilumab (anti-IL-4/IL-13): This monoclonal antibody targets both interleukin-4 (IL-4) and interleukin-13 (IL-13), which are key cytokines involved in the type 2 inflammatory pathway in asthma. Dupilumab is approved for use in children aged 6 years and older with moderate-to-severe asthma with an eosinophilic phenotype or with oral corticosteroid-dependent asthma.

These biologic therapies can be used as add-on treatments to conventional asthma medications, such as inhaled corticosteroids and bronchodilators, to help improve asthma control and reduce the risk of exacerbations in children with uncontrolled severe asthma.

mab (Nucala):

Target: Interleukin-5 (IL-5).

Mechanism: IL-5 is a cytokine that promotes the production and survival of eosinophils, a type of white blood cell involved in airway inflammation in some types of asthma. Mepolizumab reduces eosinophil levels, thereby reducing inflammation.

Indication in Children: Approved for children 6 years and older with severe eosinophilic asthma. This is often diagnosed based on blood eosinophil counts.

Administration: Subcutaneous injection, usually given every 4 weeks.

Reslizumab (Cinqair):

Target: Interleukin-5 (IL-5).

Mechanism: Similar to mepolizumab, reslizumab also targets IL-5 to reduce eosinophil levels and inflammation.

Indication in Children: Approved for children 18 years and older with severe eosinophilic asthma. While technically approved for children, it’s generally used in older adolescents and adults.

Administration: Intravenous infusion, usually given every 4 weeks.

Benralizumab (Fasenra):

Target: IL-5 receptor alpha subunit.

Mechanism: Benralizumab targets the receptor for IL-5 on eosinophils, leading to direct depletion of eosinophils through antibody-dependent cell-mediated cytotoxicity. This results in a more profound reduction in eosinophils compared to mepolizumab and reslizumab.

Indication in Children: Approved for children 6 years and older with severe eosinophilic asthma.

Administration: Subcutaneous injection, usually given every 4 weeks for the first 3 doses, then every 8 weeks thereafter.

Dupilumab (Dupixent):

Target: Interleukin-4 receptor alpha subunit (IL-4Rα), blocking signaling of both IL-4 and IL-13.

Mechanism: IL-4 and IL-13 are key cytokines involved in type 2 inflammation, which is a common pathway in asthma, including both allergic and eosinophilic asthma. Dupilumab broadly reduces type 2 inflammation.

Indication in Children: Approved for children 6 years and older with moderate-to-severe asthma with an eosinophilic phenotype or with oral corticosteroid-dependent asthma. It’s also approved for children with atopic dermatitis and chronic rhinosinusitis with nasal polyps, conditions that often co-exist with asthma and are driven by type 2 inflammation.

Administration: Subcutaneous injection, frequency varies depending on age and weight.

Tezepelumab (Tezspire):

Target: Thymic stromal lymphopoietin (TSLP).

Mechanism: TSLP is an upstream cytokine that initiates multiple inflammatory pathways involved in asthma. By blocking TSLP, tezepelumab can reduce a broader range of inflammation compared to biologics targeting downstream cytokines like IL-5 or IgE.

Indication in Children: Approved for children 12 years and older with severe asthma. It is approved for severe asthma regardless of phenotype (allergic or eosinophilic).

Administration: Subcutaneous injection, given every 4 weeks.

Important Considerations:

Diagnosis and Phenotype: The choice of biologic therapy depends on the specific type (phenotype) of severe asthma the child has. For example, omalizumab is for allergic asthma, while mepolizumab, reslizumab, and benralizumab are for eosinophilic asthma. Dupilumab and tezepelumab have broader applications in type 2 inflammation and severe asthma respectively.

Age and Approval: It’s crucial to note the age approvals for each medication, as they vary.

Specialist Consultation: Biologic therapies are prescribed and managed by specialists, typically allergists, pulmonologists, or pediatric pulmonologists. A thorough evaluation is necessary to determine if a child is a candidate for biologic therapy and which one is most appropriate.

Add-on Therapy: Biologics are add-on therapies. They are used in conjunction with standard asthma medications, not as replacements for them.

Monitoring and Effectiveness: Once started on a biologic, children need to be monitored for effectiveness and potential side effects.

This list provides an overview of the main biologic therapies currently available for children with uncontrolled severe asthma. It is essential to consult with a healthcare professional to determine the most appropriate treatment plan for an individual child.

GPT-4o

Some add-on biologic therapies for children with uncontrolled severe asthma include:

Omalizumab (Xolair): This is an anti-IgE monoclonal antibody that helps reduce allergic reactions, commonly used in children aged 6 years and older with moderate to severe allergic asthma.

Mepolizumab (Nucala): An anti-IL-5 monoclonal antibody, approved for children aged 6 years and older. It works by reducing the number of eosinophils, which are involved in asthma inflammation.

Benralizumab (Fasenra): Also an anti-IL-5 monoclonal antibody, it targets the IL-5 receptor and reduces eosinophils. It is typically used for children aged 12 and older.

Dupilumab (Dupixent): This biologic targets the IL-4 and IL-13 pathways and is approved for children aged 6 years and older with moderate to severe asthma, particularly in those with an eosinophilic phenotype or those with a history of allergies.

Tezepelumab (Tezspire): An anti-TSLP (thymic stromal lymphopoietin) monoclonal antibody, which is used for asthma with a broader mechanism of action, inhibiting airway inflammation.

These therapies are typically prescribed when standard asthma treatments (like inhaled corticosteroids and bronchodilators) are insufficient to control symptoms. However, a healthcare professional should evaluate the suitability and dosing for individual cases.

Deepseek

Here are some add-on biologic therapies for children with uncontrolled severe asthma, based on FDA approvals and clinical evidence:

Omalizumab (Xolair®)

Approved for children ≥6 years with allergic (IgE-mediated) asthma12510.

Mepolizumab (Nucala®)

Approved for children ≥6 years with severe eosinophilic asthma124.

Dupilumab (Dupixent®)

Approved for children ≥6 years with type 2 inflammation (e.g., elevated eosinophils or FeNO)1268.

Benralizumab (Fasenra®)

Approved for adolescents ≥12 years with eosinophilic asthma1348.

Tezepelumab (Tezspire®)

Approved for adolescents ≥12 years with severe asthma regardless of biomarker status184.

Key Notes:

Omalizumab has the strongest evidence for efficacy in younger children5.

Benralizumab and mepolizumab target eosinophilic inflammation and reduce exacerbations34.

Dupilumab improves lung function and reduces exacerbations in children with Th2-high asthma.

Question8:Will the risk of exacerbation increase when a child switch from Maintenance-and-reliever therapy(MART) to conventional lCS-LABA plus as-needed SABA ?

Claude-3 Haiku

Yes, the risk of exacerbation may increase when a child switches from Maintenance-and-Reliever Therapy (MART) to conventional ICS-LABA plus as-needed SABA.

MART, also known as Single Inhaler Therapy (SIT), is a treatment approach where a single inhaler containing both an inhaled corticosteroid (ICS) and a long-acting beta-agonist (LABA) is used for both maintenance and relief of symptoms. This approach has been shown to be effective in reducing the risk of exacerbations in children with asthma.

In contrast, the conventional approach involves the use of a separate ICS-LABA inhaler for maintenance therapy and a short-acting beta-agonist (SABA) inhaler for as-needed relief of symptoms.

When a child switches from the MART approach to the conventional approach, they may lose the benefits of the integrated maintenance and relief provided by the single inhaler. This can lead to a higher risk of exacerbations, as the child may not be as consistent in using the separate inhalers or may not have the same level of control over their symptoms.

Additionally, the transition between the two treatment approaches may disrupt the child’s established asthma management routine, which can also contribute to an increased risk of exacerbations.

It is important for healthcare providers to carefully monitor the child’s asthma control and symptoms when transitioning between these treatment approaches, and to provide appropriate guidance and support to the child and their caregivers to ensure a smooth transition and maintain optimal asthma management.

Gemini 2.0 Thinking

Yes, it is possible that the risk of exacerbation could increase when a child switches from Maintenance-and-Reliever Therapy (MART) to conventional ICS-LABA plus as-needed SABA. Here’s why:

Understanding the Therapies and the Potential Difference in Exacerbation Risk:

MART (Maintenance-and-Reliever Therapy):

Uses a single inhaler containing both an inhaled corticosteroid (ICS) and a long-acting beta-agonist (LABA).

The same inhaler is used daily for maintenance to control underlying inflammation and prevent symptoms.

Crucially, it’s also used “as needed” as a reliever. This means when symptoms worsen, the child takes extra puffs of the same inhaler. This provides both bronchodilation (from the LABA) and an increased dose of ICS to address the underlying inflammation that often drives exacerbations.

Conventional ICS-LABA plus as-needed SABA:

Uses two separate inhalers.

ICS-LABA inhaler: Taken daily for maintenance to control inflammation and prevent symptoms.

SABA (Short-Acting Beta-Agonist) inhaler (like albuterol): Used “as needed” for quick relief of acute symptoms. SABAs primarily relax the airways (bronchodilation) but have minimal effect on the underlying inflammation.

Why Exacerbation Risk Might Increase with the Switch:

Reduced ICS Exposure During Symptom Worsening:

MART: When symptoms worsen and the child uses their MART inhaler as a reliever, they are getting an extra dose of ICS along with the LABA. This helps to address the inflammatory component of the exacerbation early on.

Conventional Therapy: When symptoms worsen, the child uses a SABA. While the SABA provides quick relief of bronchospasm, it does not increase the ICS dose. The underlying inflammation that might be driving the exacerbation is not being directly addressed with increased ICS at that critical time.

Potential for Under-treatment of Inflammation:

Over time, relying solely on SABA for symptom relief without increasing ICS during worsening symptoms could lead to a build-up of inflammation in the airways. This chronic inflammation makes the airways more sensitive and reactive, increasing the likelihood of future exacerbations.

Dependence on SABA Alone for Reliever:

Conventional therapy relies on SABA as the sole reliever. While effective for immediate bronchodilation, overuse of SABA (which can happen if symptoms are not well controlled) is associated with poorer asthma control and increased exacerbation risk.

Important Considerations:

Asthma Severity: The impact of switching might be more pronounced in children with more severe or poorly controlled asthma. Children with milder, well-controlled asthma on MART might be less likely to experience a significant increase in exacerbations when switched.

Adherence to Daily ICS-LABA: For conventional therapy to be effective, consistent daily use of the ICS-LABA inhaler is crucial. If adherence is poor, the risk of exacerbation will be higher regardless of whether the child was previously on MART.

Individual Patient Response: Asthma management is individualized. Some children might do well on conventional therapy, while others may experience better control and fewer exacerbations with MART.

Reasons for Switching: The reason for switching from MART to conventional therapy is important. If the switch is due to specific clinical reasons (e.g., side effects, patient preference, or specific guidelines in certain situations), the potential risks and benefits should be carefully weighed by a healthcare professional.

Conclusion:

Switching from MART to conventional ICS-LABA plus as-needed SABA could potentially increase the risk of exacerbation in some children, primarily due to the reduced ICS exposure during symptom worsening and the reliance on SABA alone for reliever medication.

It is crucial that any switch in asthma medication, especially from MART to conventional therapy, is done under the guidance and supervision of a healthcare professional. They will assess the child’s individual asthma control, risk factors, and reasons for considering the switch, and provide appropriate monitoring and adjustments to minimize the risk of exacerbations. Regular follow-up and asthma action plans are essential to manage asthma effectively regardless of the chosen therapy.

GPT-4o

Switching from Maintenance-and-Reliever Therapy (MART) to conventional LCS-LABA (Long-acting Corticosteroid - Long-acting Beta Agonist) plus as-needed SABA (Short-acting Beta Agonist) could potentially increase the risk of exacerbation in a child, depending on several factors.

Effectiveness of MART: MART combines a maintenance medication (ICS-LABA) with a reliever medication in one inhaler, providing both long-term control and immediate relief. This integrated approach allows for better symptom control, particularly for children with asthma. MART also helps prevent exacerbations by ensuring the reliever medication is used in a timely manner, potentially reducing the need for additional treatments.

Use of ICS and LABA separately: When switching to conventional LCS-LABA plus as-needed SABA, the child may no longer have the benefit of the same quick-acting, combination therapy. As-needed SABA may not be as effective in controlling sudden exacerbations as the MART approach, where the combination provides faster and more reliable relief. The child may also be less consistent in using the as-needed SABA as the primary reliever, leading to inadequate symptom management.

Adherence and technique: Switching treatments can lead to challenges in adherence, especially if the child and caregivers are not fully familiar with the new regimen. Proper technique and consistency in using both the maintenance and reliever medications are crucial to preventing exacerbations.

In summary, there could be a higher risk of exacerbation when switching from MART to conventional LCS-LABA plus as-needed SABA, as MART offers a more streamlined, integrated approach to asthma management that reduces the chance of missing doses or delaying relief during an exacerbation. Close monitoring and adjustment of the treatment plan are important in managing this risk effectively.

Deepseek

Based on current evidence and guidelines, switching a child from Maintenance-and-Reliever Therapy (MART) to conventional ICS-LABA plus as-needed SABA may increase the risk of asthma exacerbation, supported by the following points:

MART’s Superiority in Reducing Severe Exacerbations

MART (ICS-formoterol) provides both maintenance and symptom relief, delivering additional anti-inflammatory ICS during exacerbations. Studies show that MART significantly reduces severe exacerbation risks compared to fixed-dose ICS-LABA with SABA. For example, SMART strategies (similar to MART) lower severe acute exacerbation risks by increasing ICS doses during symptom flare-ups 3. In children aged 6–11 years, MART reduces severe exacerbations by 66% compared to conventional ICS-LABA regimens

SABA Overuse and Lack of Anti-Inflammatory Protection

Conventional ICS-LABA with SABA relies on SABA for symptom relief, which lacks anti-inflammatory effects. Overuse of SABA (≥3 canisters/year) is linked to increased exacerbation risks 7. Guidelines emphasize that SABA-only relief may mask worsening inflammation and lead to delayed treatment, heightening exacerbation risks 67.

Guideline Recommendations

GINA guidelines discourage SABA monotherapy and recommend MART for high-risk patients to ensure continuous ICS coverage during exacerbations 69. For children, long-term SABA use may also cause tolerance and delayed response to rescue therapy 7.

Conclusion: Transitioning from MART to ICS-LABA + SABA removes the dual anti-inflammatory and bronchodilator benefits of ICS-formoterol during exacerbations, increasing the risk of uncontrolled inflammation and severe attacks. Current evidence and guidelines 23679 support maintaining MART or other ICS-containing rescue therapies in pediatric asthma management. Any regimen change should involve careful clinical evaluation.

Question9:Will a child be addicted to asthma medications if he uses them frequently?

Claude-3 Haiku

No, children who use asthma medications frequently are not considered to be addicted to them. Here are a few key points about asthma medications and addiction:

Asthma medications like bronchodilators (e.g. albuterol) and inhaled corticosteroids are not addictive. They do not cause the same type of psychological or physical dependence that occurs with addictive drugs.

These medications are used to control asthma symptoms and prevent asthma attacks. When used as directed by a doctor, they are safe and effective for managing asthma in children.

Frequent use of asthma medications may indicate that a child’s asthma is not well-controlled. This would prompt the doctor to adjust the treatment plan, not discontinue the medications.

Stopping asthma medications abruptly can lead to worsening of asthma symptoms, but this is not the same as addiction. The child’s body is not craving the medication, it’s just that the asthma is no longer being properly managed.

The key is to use asthma medications as prescribed by the child’s doctor. This ensures the medications are being used appropriately to control asthma, not out of addiction or dependence. Proper management of a child’s asthma is important to prevent complications.

Gemini 2.0 Thinking

Generally, no, a child will not become addicted to asthma medications in the way we typically think of addiction (like drug addiction).

Here’s a breakdown to explain why and address some nuances:

Understanding “Addiction” vs. “Dependence” vs. “Tolerance” in this context:

Addiction (Psychological Dependence): This involves compulsive drug-seeking behavior, craving, and continued use despite negative consequences. Asthma medications do not typically cause this type of psychological addiction. They don’t produce the same kind of “high” or reward pathway activation in the brain that addictive substances do.

Physical Dependence: This occurs when the body adapts to a medication, and withdrawal symptoms occur if the medication is stopped abruptly. While some medications can cause physical dependence, asthma medications are not known to cause significant physical dependence in the addictive sense. Stopping them suddenly might lead to a return of asthma symptoms, but this is not withdrawal from the medication itself, but rather the underlying asthma flaring up again.

Tolerance: This means the medication becomes less effective over time, requiring higher doses to achieve the same effect. Tolerance can develop to some asthma medications, particularly rescue inhalers (like albuterol). This is not addiction, but it can lead to a child needing to use their rescue inhaler more frequently, which can be concerning and needs to be addressed by a doctor.

Types of Asthma Medications and Addiction Potential:

It’s important to distinguish between different types of asthma medications:

Rescue Inhalers (Short-acting Beta-Agonists like Albuterol): These are used to quickly relieve asthma symptoms like wheezing, coughing, and shortness of breath.

Not Addictive: They are not addictive in the psychological or physical dependence sense.

Potential for Overuse: If a child is using their rescue inhaler very frequently (more than twice a week, or needing it daily), it’s a sign that their asthma is not well-controlled. This is not addiction, but rather an indication that their controller medication needs to be adjusted or that triggers need to be better managed. Overuse can also lead to side effects like increased heart rate and shakiness, and in the long run, may even worsen asthma control if underlying inflammation isn’t addressed.

Tolerance: As mentioned, tolerance can develop, making them less effective over time if used too frequently.

Controller Medications (Inhaled Corticosteroids, Leukotriene Modifiers, Long-acting Beta-Agonists, etc.): These are taken daily to prevent asthma symptoms and reduce airway inflammation.

Not Addictive: These medications are not addictive at all. They work to treat the underlying cause of asthma and are crucial for long-term management.

No Withdrawal: Stopping them abruptly might lead to a return of asthma symptoms, but this is not withdrawal from the medication itself. It’s simply the asthma symptoms returning because the medication is no longer controlling the inflammation.

Why Frequent Use of Rescue Inhalers is a Concern (But Not Addiction):

If a child is using their rescue inhaler frequently, it’s a sign that:

Their asthma is not well-controlled. They likely need better controller medication or a review of their asthma management plan.

They might be exposed to triggers. Identifying and avoiding triggers is crucial for asthma control.

They might be developing tolerance to the rescue inhaler.

What to Do If You’re Concerned About Frequent Asthma Medication Use:

Talk to your child’s doctor. Frequent use of rescue inhalers is a medical issue that needs to be addressed. The doctor can assess your child’s asthma control, adjust medications, and provide guidance on managing triggers.

Follow the doctor’s prescribed asthma action plan. This plan will outline when to use rescue inhalers and controller medications.

Don’t stop asthma medications without consulting a doctor. This can be dangerous and lead to worsening asthma symptoms.

In summary:

Asthma medications, both rescue and controller medications, are not addictive in the way drugs of abuse are. Frequent use of rescue inhalers is a sign of poorly controlled asthma, not addiction.It’s crucial to work with your child’s doctor to ensure their asthma is well-managed and they are using their medications appropriately.

GPT-4o

Asthma medications, especially inhalers, are typically not considered addictive in the way substances like alcohol or drugs are. However, it’s important to use them as prescribed by a healthcare provider to manage asthma symptoms effectively. Overuse or misuse can sometimes lead to side effects or complications.

There are two main types of asthma medications:

Rescue inhalers (bronchodilators) – These are used to relieve acute asthma symptoms or attacks by opening the airways. If a child is frequently using a rescue inhaler, it may indicate that their asthma is not well-controlled, and they should be evaluated by a doctor to adjust their treatment plan.

Controller medications (steroids or long-acting bronchodilators) – These are taken regularly to reduce inflammation and prevent asthma symptoms. When used as directed, they help prevent asthma attacks but don’t cause addiction.

Frequent use of a rescue inhaler might suggest the asthma isn’t being managed well and may result in side effects, such as increased heart rate or jitteriness, rather than addiction.

It’s important to consult with a pediatrician to ensure that the treatment plan is optimal and that the asthma is under control. If a child needs to use their inhaler more often than usual, it could be a sign that the condition needs reevaluation.

Deepseek

No, a child will not become addicted to asthma medications when used as prescribed by a healthcare provider. However, there are important nuances to understand based on the provided references and medical evidence:

1. Asthma medications are not addictive

Most asthma medications, such as inhaled corticosteroids (e.g., budesonide) and short-acting beta-agonists (e.g., albuterol), do not cause psychological addiction. They are designed to reduce airway inflammation or relieve symptoms, not to induce compulsive drug-seeking behavior.

Studies confirm that children using asthma medications as prescribed show no increased risk of substance abuse compared to non-users.

2. Physiological dependence vs. addiction

Dependence (reliance on medication to manage symptoms) may occur if asthma is poorly controlled,leading to frequent use of rescue inhalers like albuterol. This is not addiction but reflects the need for better symptom management.

Addiction involves harmful, compulsive use for non-medical purposes. While rare, inhaler misuse (e.g., using them to “get high”) has been reported in adolescents with preexisting antisocial behavior or substance abuse issues.

3. Risks of overuse

Overusing rescue inhalers (e.g., exceeding prescribed doses) may indicate poor asthma control, not addiction. It can lead to side effects like increased heart rate or tremors but does not cause dependency on the drug itself.

Long-term use of inhaled corticosteroids at prescribed doses is safe for children, though high doses may rarely slow growth or cause oral thrush.

4. Key recommendations

Follow the prescribed treatment plan and use a spacer with inhalers for proper delivery57.

Regular check-ups with a pediatrician or pulmonologist are crucial to adjust medication and ensure asthma is well-controlled58.

Address concerns about side effects (e.g., weight gain, bone health) with a doctor, as these are manageable and unrelated to addiction.

Conclusion

Properly managed asthma medications are safe and non-addictive. If a child requires frequent use of rescue inhalers, consult a healthcare provider to reassess the treatment plan rather than assuming addiction.

Question10:After an asthmatic exacerbation,How soon should a review visit be scheduled?What does the frequency of visit depend on?

Claude-3 Haiku

After an asthmatic exacerbation, a review visit should be scheduled as soon as possible, typically within 2-4 weeks. The frequency of the follow-up visits depends on several factors:

Severity of the exacerbation: Patients who have experienced a severe exacerbation, requiring hospitalization or the use of oral corticosteroids, may need more frequent follow-up visits, such as every 2-4 weeks, to ensure proper management and monitoring of their condition.

Asthma control: The frequency of visits also depends on the level of asthma control. Patients with poorly controlled asthma, who continue to experience symptoms or require frequent use of rescue medications, may need more frequent follow-up visits, such as every 4-8 weeks, to adjust their treatment plan and achieve better control.

Adherence to treatment: Patients who have difficulty adhering to their prescribed asthma medications or treatment plan may require more frequent visits to address any barriers to adherence and ensure proper management of their condition.

Presence of comorbidities: Patients with other underlying medical conditions, such as allergies, sinusitis, or gastroesophageal reflux disease (GERD), may need more frequent follow-up visits to manage the interactions between these conditions and their asthma.

Patient preference and needs: The frequency of visits may also be influenced by the patient’s preferences, concerns, and specific needs, as well as the healthcare provider’s assessment of the patient’s overall health and the need for closer monitoring.

In general, the goal is to schedule follow-up visits at a frequency that allows for close monitoring, adjustment of treatment as needed, and the achievement of optimal asthma control for the individual patient.

Gemini 2.0 Thinking

After an asthma exacerbation, a review visit should be scheduled relatively soon, typically within a few days to a week.

The frequency of these review visits depends on several factors:

1. Severity of the Exacerbation:

Severe Exacerbation (requiring Emergency Room or Hospitalization): A follow-up visit is crucial within a few days to a week after discharge. These exacerbations indicate a significant loss of asthma control, and close monitoring is needed to ensure recovery and prevent recurrence. More frequent visits might be needed initially.

Moderate Exacerbation (managed in urgent care or doctor’s office): A review visit is still important within a week. Even if managed outside the hospital, a moderate exacerbation signifies a need to reassess asthma control and adjust management.

Mild Exacerbation (managed at home): A review visit should be scheduled within 1-2 weeks. While milder, it still indicates a need to review the asthma action plan and ensure optimal control.

2. Patient’s Recovery and Symptom Control:

Persistent Symptoms: If the patient is still experiencing symptoms like cough, wheezing, shortness of breath, or chest tightness after the exacerbation, more frequent visits are needed to monitor progress and adjust treatment until symptoms are well-controlled.

Return to Baseline: If the patient has quickly returned to their baseline level of asthma control and is symptom-free, the initial follow-up might be sufficient, but a subsequent visit to assess long-term control is still important.

3. Changes in Asthma Medications:

New Medications or Dosage Adjustments: If medications were started or dosages were increased during the exacerbation, a follow-up visit is essential to assess the effectiveness of these changes, monitor for side effects, and ensure the patient is using the medications correctly.

Step-Down in Medications: Once asthma is well-controlled, the healthcare provider will consider stepping down medications to the lowest effective dose. Follow-up visits are needed to monitor for any recurrence of symptoms during this step-down process.

4. Level of Asthma Control Before the Exacerbation:

Poorly Controlled Asthma: If the patient’s asthma was poorly controlled before the exacerbation, more frequent follow-up visits are necessary to address underlying issues contributing to poor control, such as inhaler technique, adherence to medication, trigger identification and avoidance, and co-existing conditions.

Well-Controlled Asthma: Even in patients with previously well-controlled asthma, an exacerbation warrants a review to identify potential triggers or factors that led to the event and to reinforce self-management strategies.

5. Patient Education and Self-Management Skills:

Need for Reinforcement: Follow-up visits are crucial opportunities to reinforce asthma education, including inhaler technique, understanding their asthma action plan, recognizing early warning signs of exacerbations, and trigger avoidance.

Development of Asthma Action Plan: If the patient doesn’t have an asthma action plan or needs it updated, the follow-up visit is the ideal time to create or revise it.

6. Presence of Comorbidities:

Other Health Conditions: Conditions like allergies, sinusitis, GERD, or obesity can impact asthma control. Follow-up visits may need to be more frequent to manage these comorbidities and their influence on asthma.

In summary, the initial review visit after an asthma exacerbation should be scheduled within a few days to a week. The frequency of subsequent visits is then tailored to the individual patient based on the severity of the exacerbation, their recovery, symptom control, medication adjustments, pre-exacerbation asthma control, educational needs, and presence of other health conditions. The goal of these follow-up visits is to ensure the patient returns to their best possible lung function, prevent future exacerbations, and optimize long-term asthma management.

It’s important to remember that this is a general guideline, and the specific timing and frequency of follow-up visits should be determined by the healthcare provider based on the individual patient’s needs and clinical judgment.

GPT-4o

After an asthmatic exacerbation, a follow-up review visit should generally be scheduled within 1 to 2 weeks after the event. This allows healthcare providers to assess how well the patient is recovering, evaluate the effectiveness of the treatment plan, and make any necessary adjustments to their asthma management.

The frequency of visits depends on several factors:

Severity of the exacerbation: More severe exacerbations may require more frequent visits to monitor progress.

Response to treatment: If the patient is not responding well to treatment, more frequent follow-ups may be necessary.

Patient’s baseline asthma control: If asthma was poorly controlled before the exacerbation, closer monitoring might be needed to adjust long-term management strategies.

Comorbidities: Patients with other health conditions might require more frequent monitoring.

Age of the patient: Younger or older patients may need additional attention due to variations in how asthma affects them.

Overall, the goal is to ensure that asthma is well-controlled and to prevent further exacerbations.

Deepseek

After an asthma exacerbation, a review visit should ideally be scheduled within 1 week to assess treatment response, adjust medications, and prevent future episodes78.

The frequency of follow-up visits depends on:

Disease severity and control: Poorly controlled asthma or severe cases may require more frequent visits (e.g., every 1–3 months)13.

Recent treatment adjustments: Step-up or step-down therapy warrants closer monitoring (1–3 months)1.

Risk of recurrence: Patients with a history of frequent exacerbations or emergency visits may need 3-monthly follow-ups36.

Patient adherence and education: Regular visits ensure proper inhaler technique and adherence to action plans58.

Comorbidities: Conditions like allergies or chronic rhinosinusitis may necessitate additional evaluations9.

For stable asthma, maintenance follow-ups every 3–12 months are generally recommended17. Post-exacerbation care should prioritize early review to reduce relapse risk

Question11:If a child is exposed to viral infections or seasonal allergen exposure,Is a short-term increase in maintenance lCS dose for 1-2 weeks be necessary?

Claude-3 Haiku

Yes, a short-term increase in the maintenance inhaled corticosteroid (ICS) dose for 1-2 weeks may be necessary if a child is exposed to viral infections or seasonal allergen exposure.

Here’s why:

Viral infections:

Viral respiratory infections, such as the common cold or flu, can trigger inflammation in the airways and worsen asthma symptoms.

Increasing the ICS dose temporarily can help reduce this inflammation and prevent further exacerbation of asthma symptoms.

The increased ICS dose is typically recommended for 1-2 weeks, or until the viral infection has resolved and the child’s asthma symptoms have stabilized.

Seasonal allergen exposure:

Exposure to seasonal allergens, such as pollen, can cause an increase in airway inflammation and worsen asthma symptoms.

Increasing the ICS dose temporarily can help control the inflammatory response and prevent asthma exacerbations.

The increased ICS dose is typically recommended for 1-2 weeks, or until the peak of the allergy season has passed and the child’s asthma symptoms have stabilized.

It’s important to note that the specific dose increase and duration of the increased ICS dose should be determined by the child’s healthcare provider, based on the individual’s asthma severity, symptoms, and response to the increased medication.

Gemini 2.0 Thinking

Yes, in some cases, a short-term increase in the maintenance inhaled corticosteroid (ICS) dose for 1-2 weeks can be necessary when a child is exposed to viral infections or seasonal allergen exposure, especially if they have asthma. However, it’s not a blanket recommendation for every child in every situation.

Here’s a more detailed explanation:

Reasons why a short-term increase might be considered:

Viral Infections: Viral respiratory infections (like colds and flu) are a very common trigger for asthma exacerbations in children. Viruses cause inflammation in the airways, which can worsen asthma symptoms like wheezing, coughing, and shortness of breath. Increasing the ICS dose temporarily can help to counteract this increased inflammation and prevent a full-blown asthma flare-up.

Seasonal Allergen Exposure: For children with allergic asthma, exposure to seasonal allergens like pollen, mold, or dust mites can also trigger airway inflammation and asthma symptoms. A short-term increase in ICS during peak allergen seasons or when exposure is known to be high might help to preemptively control inflammation.

Proactive Management: The goal of increasing the ICS dose in these situations is often to be proactive and prevent symptoms from worsening to the point where oral corticosteroids (like prednisone) are needed. Oral steroids have more potential side effects than inhaled corticosteroids, so avoiding them is generally preferred if possible.

Individualized Asthma Action Plan: Many children with asthma have a written asthma action plan developed with their doctor. This plan often includes instructions on when and how to adjust medications, including potentially increasing the ICS dose for short periods during illness or allergen exposure.

Important Considerations:

Not Always Necessary: Not every child with asthma will need an ICS dose increase for every viral infection or allergen exposure. Many children can manage with their usual maintenance dose and increased use of their reliever medication (like albuterol) as needed.

Severity of Asthma: Children with more severe or poorly controlled asthma are more likely to benefit from a short-term ICS dose increase during triggers.

Symptoms: The decision to increase the ICS dose should be based on the child’s symptoms. If they are experiencing worsening cough, wheezing, increased use of their reliever medication, or difficulty breathing, an increase might be appropriate.

Doctor’s Advice is Crucial: It is essential to discuss this strategy with the child’s doctor or healthcare provider. They can assess the child’s individual situation, asthma severity, usual triggers, and response to treatment to determine if a short-term ICS dose increase is appropriate and provide specific instructions. Do not increase the ICS dose without consulting a doctor, especially for the first time.

Duration: The increase is typically short-term, usually for 1-2 weeks, or as directed by the doctor. Long-term increases in ICS dose should only be done under medical supervision.

Reliever Medication: Even with a short-term ICS increase, the child should still use their reliever medication (like albuterol) as needed for quick relief of acute symptoms.

Oral Corticosteroids May Still Be Needed: In some cases, despite increasing the ICS dose, the child may still develop a more severe asthma exacerbation requiring oral corticosteroids.

In summary:

A short-term increase in maintenance ICS dose for 1-2 weeks can be a helpful strategy for some children with asthma when exposed to viral infections or seasonal allergens, particularly if they are prone to exacerbations. However, it is not a universal recommendation and should be individualized and guided by a doctor’s advice. Always consult with a healthcare professional to determine the best management plan for your child’s asthma.

GPT-4o

Yes, a short-term increase in the maintenance inhaled corticosteroid (ICS) dose for 1-2 weeks may be necessary when a child is exposed to viral infections or seasonal allergen exposure. This adjustment is commonly done to prevent an asthma exacerbation or control worsening symptoms. The increase in ICS dose can help reduce inflammation in the airways and prevent flare-ups. However, it is important to follow the guidance of a healthcare professional to ensure proper dosing and duration of the adjustment.

Deepseek

Based on the available evidence and guidelines, a short-term increase in maintenance inhaled corticosteroid (ICS) dose for 1–2 weeks may be considered in children exposed to viral infections or seasonal allergens, particularly if they experience worsening asthma symptoms or enter the “yellow zone” (indicating a risk of exacerbation). However, the evidence supporting this approach is mixed, and recommendations vary by age and clinical context:

For children <6 years old:

Intermittent high-dose ICS during viral infections may reduce exacerbations by 35% 3.

Short-term step-up in ICS dose during infections is occasionally recommended, though evidence is limited 7.

For school-aged children and adults:

Quadrupling the maintenance ICS dose for 7–14 days during early signs of exacerbation (e.g., triggered by allergens or viruses) has shown some benefit in older children and adults 510. However, this exceeds product monograph limits in some cases and requires caution 10.

General considerations:

Guidelines acknowledge that individual responsiveness to ICS varies, and short-term dose adjustments may help in uncontrolled cases 47.

Long-term or excessive ICS dose escalation between exacerbations is not recommended for children with well-controlled asthma 6.

Practical recommendation:

If a child has a history of viral/allergen-triggered exacerbations, a temporary ICS dose increase (e.g., doubling or quadrupling) for 1–2 weeks during high-risk periods (e.g., viral season or allergen exposure) could be justified, especially if supported by their asthma action plan. However, this should be individualized and balanced against potential side effects 1710. Always follow local guidelines and consult clinical protocols for age-specific dosing limits.

2

Question12:When you know that you are going to be exposed to something that triggers your asthma, can I take medication just before exposure to prevent asthma?

Claude-3 Haiku

Yes, you can take medication before exposure to something that triggers your asthma in order to prevent an asthma attack. This is known as “pre-treatment” or “pre-medication.”

Some common medications that can be used for this purpose include:

Bronchodilators (e.g., albuterol, levalbuterol): These medications work quickly to relax and open the airways, making it easier to breathe. They can be taken 15-30 minutes before exposure to help prevent asthma symptoms.

Inhaled corticosteroids (e.g., fluticasone, budesonide): These medications reduce inflammation in the airways and can be taken daily or before exposure to help prevent asthma symptoms.

Leukotriene modifiers (e.g., montelukast, zafirlukast): These medications block the action of leukotrienes, which are chemicals that can trigger asthma symptoms. They can be taken daily or before exposure to help prevent asthma symptoms.

It’s important to discuss your specific asthma triggers and treatment options with your healthcare provider to determine the best pre-treatment strategy for your needs. They can provide guidance on the appropriate medication, dosage, and timing to effectively prevent asthma symptoms before exposure.

Gemini 2.0 Thinking

Yes, in many cases, you can take medication just before exposure to a known asthma trigger to help prevent an asthma attack. This is a common and effective strategy for managing asthma, but it’s crucial to understand which medications are appropriate and how to use them correctly.

Here’s a breakdown of what you need to know:

Types of Medications Used Before Exposure:

Quick-Relief Inhalers (Rescue Inhalers): These are the most common type of medication used for pre-exposure prevention. They contain short-acting beta-agonists (SABAs) like albuterol (Ventolin, ProAir, Proventil).

How they work: SABAs quickly relax the muscles around your airways, opening them up and making it easier to breathe.

When to take them: Typically, you should take your rescue inhaler 15-20 minutes before you expect to be exposed to your trigger. This allows the medication to start working before the trigger can cause your airways to narrow.

Examples of triggers where this is helpful:

Exercise-induced asthma: Taking a puff or two before exercise can prevent symptoms.

Exposure to cold air: Using it before going outside in cold weather.

Known allergen exposure (e.g., pet dander, dust mites, pollen): If you know you’ll be in a situation with these allergens.

Exposure to irritants (e.g., strong fumes, smoke): If you anticipate being around these.

Important Note: While rescue inhalers are effective for short-term prevention, they are not a substitute for long-term control medications if you need them. Over-reliance on rescue inhalers can indicate that your asthma is not well-controlled and you need to discuss a better management plan with your doctor.

Other Medications (Less Common for Immediate Pre-Exposure, but Important for Overall Control):

Inhaled Corticosteroids (ICS): These are long-term control medications that reduce inflammation in your airways. They are taken daily to prevent asthma symptoms from occurring in the first place. While you don’t take them just before exposure for immediate relief, consistent use of ICS makes you less reactive to triggers and reduces the likelihood of needing rescue medication.

Combination Inhalers (ICS/LABA): These combine an inhaled corticosteroid with a long-acting beta-agonist (LABA). Like ICS, they are for daily, long-term control and help reduce overall airway inflammation and prevent symptoms. They are not typically used just before exposure for immediate relief, but contribute to better overall asthma control.

Leukotriene Modifiers (e.g., montelukast, zafirlukast): These are oral medications that block the action of leukotrienes, chemicals that contribute to inflammation and airway narrowing in asthma. They are also long-term control medications taken daily and are not for immediate pre-exposure use. However, they can help reduce your overall sensitivity to triggers.

Mast Cell Stabilizers (e.g., cromolyn sodium, nedocromil): These medications prevent the release of substances that cause inflammation in the airways. They are less commonly used now, but can be used preventively, sometimes taken before exercise or allergen exposure. They need to be taken regularly for best effect.

Key Considerations and Recommendations:

Talk to your doctor: The most important step is to discuss your asthma management with your doctor. They can:

Diagnose your asthma properly.

Identify your specific triggers.

Prescribe the right medications for you.

Develop an asthma action plan that outlines when and how to use your medications, including pre-exposure use.

Teach you proper inhaler technique.

Follow your asthma action plan: This plan should be personalized to you and will guide you on when to use your medications, including pre-exposure use of your rescue inhaler.

Don’t overuse rescue inhalers: If you find yourself needing to use your rescue inhaler frequently (more than twice a week, excluding exercise-induced asthma), it’s a sign that your asthma is not well-controlled. You should discuss this with your doctor, as you may need adjustments to your long-term control medication.

Carry your rescue inhaler with you: Always have your rescue inhaler readily available, especially when you know you might be exposed to triggers.

Understand your triggers: Knowing what triggers your asthma is crucial for effective prevention.

In summary, yes, taking a rescue inhaler (short-acting beta-agonist) 15-20 minutes before exposure to a known asthma trigger is a common and effective way to prevent asthma symptoms. However, it’s essential to have a proper diagnosis, a personalized asthma action plan, and to discuss your asthma management with your doctor to ensure you are using the right medications in the right way for your specific needs.

GPT-4o

Yes, it is possible to take medication before exposure to an asthma trigger to help prevent symptoms. For instance, using a short-acting bronchodilator (like albuterol) can help open your airways and prevent asthma symptoms if taken 15-30 minutes before exposure to a trigger.

If you have a prescription for a preventive inhaler (e.g., corticosteroids), these are generally used on a regular basis rather than just before exposure. However, it’s always important to follow your doctor’s advice and treatment plan.

If you’re unsure or your asthma is not well controlled, it’s a good idea to consult your healthcare provider for specific recommendations tailored to your condition.

Deepseek

Yes, you can take certain medications before exposure to known asthma triggers to help prevent symptoms. Here’s a concise summary of the recommendations based on the references:

Short-Acting Beta-Agonists (SABAs): Medications like albuterol or levalbuterol can be used 15–30 minutes before exercise or exposure to triggers (e.g., allergens, cold air) to prevent bronchoconstriction256.

Inhaled Corticosteroids (ICS): Regular use of ICS (e.g., budesonide) reduces airway inflammation and prevents exacerbations, especially for chronic asthma management310.

Combination Inhalers: For exercise-induced asthma, ICS-long-acting beta-agonist (LABA) combinations (e.g., budesonide-formoterol) are effective when used preemptively5.

Avoidance of Triggers: Environmental control (e.g., avoiding allergens) remains a critical preventive measure14.

Always consult your healthcare provider to tailor the medication plan to your specific triggers and asthma severity.

Question13:For children aged 6-11,On what condition can the the treatment be successfully reduced?

Claude-3 Haiku

For children aged 6-11, the treatment can be successfully reduced if the child’s condition improves and they show consistent progress in managing their symptoms. The specific conditions for reducing treatment may vary depending on the child’s diagnosis and the treatment plan developed by their healthcare provider.

Some general factors that may indicate the possibility of reducing treatment include:

Symptom improvement: If the child’s symptoms have significantly improved and they are able to function well in various settings (e.g., school, home, social activities), the healthcare provider may consider reducing the intensity or frequency of the treatment.

Coping strategies development: If the child has developed effective coping strategies and self-management skills to manage their condition, the healthcare provider may feel confident in reducing the level of support or intervention.

Stable functioning: If the child has maintained a stable level of functioning for an extended period, the healthcare provider may determine that a gradual reduction in treatment is appropriate.

Parental/caregiver involvement: The healthcare provider may also consider the level of involvement and support from the child’s parents or caregivers in managing the condition. If the family is actively engaged and able to provide the necessary support, the treatment may be reduced.

It is important to note that the decision to reduce treatment should be made in close collaboration with the child’s healthcare provider, who will carefully evaluate the child’s progress, monitor their response, and make adjustments as needed to ensure the best possible outcome for the child.

Gemini 2.0 Thinking

For children aged 6-11, treatment can be successfully reduced when there is clear and sustained evidence of improvement in their condition and they are stable enough to function well with less intervention.

Here’s a breakdown of the conditions:

Demonstrated Improvement: This is the most crucial condition. The child’s symptoms or the underlying issue being treated must have demonstrably improved. This improvement should be:

Observable and Measurable: It shouldn’t just be a feeling; there needs to be objective evidence of progress. This could be through:

Reduced symptom severity: Less frequent or less intense symptoms.

Improved functioning: Better performance in school, social situations, or daily activities.

Objective tests or assessments: Improved scores on relevant tests or assessments related to their condition (e.g., allergy tests, ADHD rating scales, therapy progress reports).

Sustained: The improvement needs to be consistent over a period of time, not just a temporary fluctuation. This period will vary depending on the condition and treatment.

Stability: The child’s condition needs to be stable enough to handle a reduction in treatment without a relapse or worsening of symptoms. This means:

Underlying condition is well-managed: The treatment has effectively brought the condition under control.

Resilience: The child has developed some coping mechanisms or resilience to manage their condition with less support.

Professional Assessment and Guidance: Crucially, any decision to reduce treatment must be made in consultation with the healthcare professional overseeing the child’s care. This could be a doctor, therapist, specialist, or other qualified professional. They will:

Assess the child’s progress: Evaluate the evidence of improvement and stability.

Consider the specific condition and treatment: Different conditions and treatments have different protocols for reduction.

Develop a gradual reduction plan: Treatment reduction should usually be gradual and monitored to ensure it’s safe and effective.

Monitor for relapse: Even after reduction, ongoing monitoring is important to catch any signs of relapse early.

Individualized Approach: The decision to reduce treatment is highly individualized and depends on the specific child, their condition, their response to treatment, and their overall circumstances. There is no one-size-fits-all answer.

In summary, successful treatment reduction in children aged 6-11 hinges on demonstrable and sustained improvement, stability of their condition, and a carefully planned and monitored reduction process guided by healthcare professionals. It’s not about stopping treatment abruptly, but rather carefully and thoughtfully stepping down the level of intervention as the child’s condition improves.

GPT-4o

In children aged 6-11, the treatment can be successfully reduced under the following conditions:

Improved Symptoms: If the child shows significant improvement in symptoms and is stable over time, treatment can often be reduced.

Stable Health Condition: When the child has maintained a stable health condition without significant flare-ups or complications, the need for intensive treatment may lessen.

Proper Growth and Development: If the child is growing and developing normally, indicating that the condition is well-managed, reducing treatment may be considered.

Reduced Severity of the Condition: In cases where the child’s illness or condition has become less severe or is in remission, a doctor may deem it appropriate to lower the treatment dosage or frequency.

Positive Test Results: In some cases, if laboratory tests (such as blood tests or imaging) show improvements or stable results, this could lead to a reduction in treatment.

Doctor’s Evaluation: The decision to reduce treatment must be made by a healthcare provider, who will monitor the child’s progress and ensure that any reduction in treatment is safe.

It is important that any changes to a treatment plan be done under the guidance of a healthcare professional to ensure the child’s health and safety.

Deepseek

For children aged 6-11, treatment (especially for conditions like benign epilepsy) can be successfully reduced under the following conditions:

Sustained Symptom Control: The child must remain seizure-free for 2-3 years with no recurrence of symptoms during this period13.

Normalized Brain Activity: Electroencephalogram (EEG) results should show no epileptiform discharges or other abnormal brain activity3.

Gradual Dose Reduction: The reduction should occur slowly over 3-6 months to avoid triggering relapse. Abrupt discontinuation is not recommended13.

Age Consideration: While younger children (e.g., under 5) might require stricter criteria, children aged 6-11 can start tapering after meeting the above conditions1.

For general medication adjustments (e.g., antibiotics, fever reducers), dosage reductions are typically based on weight calculations (e.g., using formulas like

Weight (kg)=Age×2+8

Weight (kg)=Age×2+8) and clinical response246. Always consult a pediatrician for tailored guidance.

Question14:Can high baseline of FeNO been used to predict exacerbation after step-down of lCS dose?

Claude-3 Haiku

Yes, a high baseline of fractional exhaled nitric oxide (FeNO) can be used to predict the risk of exacerbation after step-down of inhaled corticosteroid (ICS) dose in patients with asthma.

FeNO is a non-invasive biomarker that reflects airway inflammation, and a high baseline FeNO level has been associated with an increased risk of asthma exacerbations. When patients with asthma are on a stable ICS regimen, a high baseline FeNO level can indicate the presence of ongoing airway inflammation, even in the absence of clinical symptoms.

When these patients undergo a step-down in their ICS dose, the high baseline FeNO level can be used to predict an increased risk of asthma exacerbations. This is because the reduced ICS dose may not be sufficient to control the underlying airway inflammation, leading to a higher likelihood of exacerbations.

Several studies have demonstrated that a high baseline FeNO level (typically defined as FeNO > 50 ppb) is associated with a higher risk of exacerbations after ICS step-down, compared to patients with lower baseline FeNO levels. This information can be useful for clinicians when making decisions about ICS step-down in patients with asthma, as it can help identify those who may be at a higher risk of experiencing an exacerbation and require closer monitoring or a more gradual step-down approach.

Gemini 2.0 Thinking

Yes, a high baseline FeNO (fractional exhaled nitric oxide) level can be used to predict an increased risk of exacerbation after step-down of inhaled corticosteroid (ICS) dose in asthma patients.

Here’s why and how:

FeNO as a Marker of Eosinophilic Airway Inflammation: FeNO is a non-invasive biomarker that reflects eosinophilic airway inflammation, a key feature in many types of asthma, particularly steroid-responsive asthma. Higher FeNO levels generally indicate more eosinophilic inflammation in the airways.

ICS and Eosinophilic Inflammation: ICS medications are effective in reducing eosinophilic airway inflammation. They are the cornerstone of asthma maintenance therapy. Stepping down ICS aims to find the lowest effective dose to control asthma symptoms and minimize potential side effects.

High Baseline FeNO Suggests Persistent Inflammation: If a patient has a high FeNO level before ICS dose reduction, it suggests that there is still significant underlying eosinophilic inflammation present in their airways, even at their current ICS dose. This indicates that their asthma may not be fully controlled at the inflammatory level, even if their symptoms are seemingly well-managed.

Increased Risk of Exacerbation After Step-Down: When ICS dose is reduced in a patient with high baseline FeNO, the anti-inflammatory effect of ICS is lessened. This can lead to a resurgence of eosinophilic inflammation, potentially triggering asthma exacerbations. The pre-existing high FeNO signals a vulnerability to this inflammatory rebound.

Clinical Evidence: Several studies have shown that baseline FeNO levels can predict exacerbations after ICS step-down. Patients with higher baseline FeNO are more likely to experience asthma worsening or exacerbations when their ICS dose is reduced compared to those with lower baseline FeNO.

Important Considerations:

FeNO is not a perfect predictor: While high baseline FeNO increases the risk, it’s not a guarantee of exacerbation. Other factors also contribute to exacerbation risk, such as adherence to medication, environmental triggers, viral infections, and individual asthma phenotype.

Context is crucial: FeNO should be interpreted in the context of the patient’s overall clinical picture, including their asthma history, symptom control, lung function, and other risk factors.

FeNO-guided step-down: FeNO can be used as a tool to guide ICS step-down decisions. In patients with low FeNO and good asthma control, step-down may be considered more safely. However, in patients with high FeNO, step-down should be approached cautiously, and closer monitoring is warranted. Some guidelines even suggest maintaining or even increasing ICS dose in patients with high FeNO despite seemingly controlled symptoms.

In summary, a high baseline FeNO level before ICS step-down is a valuable indicator of persistent eosinophilic airway inflammation and can predict an increased risk of asthma exacerbation after ICS dose reduction. It should be considered as one factor in clinical decision-making regarding ICS step-down and patient monitoring.

GPT-4o

Yes, a high baseline level of Fractional Exhaled Nitric Oxide (FeNO) has been proposed as a potential predictor of exacerbations in asthma after a step-down of inhaled corticosteroid (ICS) doses. FeNO is a biomarker that reflects airway inflammation, particularly eosinophilic inflammation, which is common in asthma.

Several studies suggest that elevated FeNO levels are associated with increased inflammation in the airways, and this can help predict the risk of asthma exacerbations, even when ICS doses are reduced. Monitoring FeNO before and after ICS dose adjustments may allow clinicians to identify patients who are at higher risk of worsening symptoms or exacerbations and need closer monitoring or further interventions.

However, while FeNO can be a useful marker, it should be used in conjunction with other clinical signs and symptoms to guide treatment decisions. It is not a sole predictor but can be a helpful tool for personalized asthma management.

Deepseek

Yes, a high baseline FeNO (fractional exhaled nitric oxide) level can be used to predict the risk of asthma exacerbation after step-down of inhaled corticosteroid (ICS) dose. Multiple studies and clinical guidelines suggest the following:

Baseline FeNO >50 ppb is strongly associated with increased exacerbation risk. For example, patients with FeNO >45–50 ppb have a nearly 6-fold higher annual exacerbation rate compared to those with lower FeNO levels12.

FeNO reflects type 2 (T2) inflammation, and persistently elevated FeNO indicates ongoing airway inflammation even under ICS therapy. Reducing ICS doses in such patients may lead to loss of control and exacerbations58.

Clinical utility: FeNO ≥25–50 ppb is recommended as a threshold to guide ICS adjustment. Lowering ICS doses in patients with FeNO <25 ppb is safer, while those with higher FeNO values (especially ≥50 ppb) should maintain or increase ICS doses to avoid exacerbations358.

Predictive accuracy: FeNO, combined with blood eosinophil counts, improves prediction of exacerbation risk after ICS reduction15.

In conclusion, monitoring FeNO before ICS step-down is clinically valuable. High baseline FeNO (>50 ppb) signals a significant risk of exacerbation, necessitating cautious dose reduction and closer monitoring

Question15:Before asthma treatment step-down,what should be evaluated by patients? Claude-3 Haiku

Before considering an asthma treatment step-down, the following factors should be evaluated by patients:

Asthma control: Patients should assess their level of asthma control, including the frequency of symptoms, use of rescue medications, and any limitations in daily activities due to asthma.

Lung function: Patients should have their lung function, measured by spirometry or peak flow, evaluated to determine the current status of their asthma.

Asthma triggers: Patients should identify and understand their specific asthma triggers, such as allergens, irritants, or environmental factors, to ensure that these are well-managed before considering a step-down in treatment.

Adherence to current treatment: Patients should evaluate their adherence to the current asthma treatment regimen, including the proper use of inhalers and other medications, to ensure that the lack of control is not due to poor adherence.

Exacerbation history: Patients should review their history of asthma exacerbations, including the frequency, severity, and any hospitalizations or emergency department visits, to determine if a step-down in treatment is appropriate.

Patient preferences and goals: Patients should discuss their personal preferences and goals for asthma management with their healthcare provider, as this can help guide the decision-making process for a potential step-down in treatment.

By evaluating these factors, patients and their healthcare providers can make an informed decision about whether a step-down in asthma treatment is appropriate and safe for the individual patient.

Gemini 2.0 Thinking

Before asthma treatment step-down, patients should be evaluated on several key aspects to ensure it’s safe and appropriate to reduce their medication. These evaluations are crucial to confirm that their asthma is well-controlled and that step-down won’t lead to a relapse of symptoms. Here’s what should be evaluated:

1. Asthma Symptom Control:

Daytime Symptoms: How often are they experiencing daytime asthma symptoms like wheezing, coughing, shortness of breath, or chest tightness? Ideally, they should have minimal to no daytime symptoms.

Nighttime Symptoms: How often are they being woken up at night due to asthma symptoms? Nighttime symptoms should also be minimal to none.

Rescue Inhaler Use: How often are they using their rescue inhaler (like albuterol)? Infrequent use (less than twice a week, excluding exercise-induced asthma) is a sign of good control. Frequent use indicates poor control.

Activity Limitation: Is their asthma limiting their normal activities, such as exercise, work, school, or sleep? Asthma should not be significantly impacting their daily life.

Asthma Control Test (ACT) or Asthma Control Questionnaire (ACQ): These are standardized questionnaires that help assess asthma control over the past few weeks. A good score on these questionnaires indicates good control.

2. Exacerbation History:

Frequency and Severity of Exacerbations: Have they had any asthma exacerbations (attacks) requiring oral corticosteroids (like prednisone) or emergency room visits in the recent past (typically within the last 3-6 months)? Being free from exacerbations for a sustained period is important before considering step-down.

3. Medication Adherence and Inhaler Technique:

Medication Adherence: Are they taking their asthma medications as prescribed consistently? Poor adherence can mimic uncontrolled asthma and needs to be addressed before step-down.

Inhaler Technique: Are they using their inhaler correctly? Incorrect technique can reduce medication effectiveness and lead to apparent poor control. This should be checked and corrected if necessary.

4. Trigger Identification and Avoidance:

Trigger Identification: Have they identified their asthma triggers (allergens, irritants, exercise, weather, etc.)?

Trigger Avoidance: Are they effectively avoiding or managing their identified triggers? Good trigger management is essential for maintaining asthma control, especially during step-down.

5. Duration of Well-Controlled Asthma:

Sustained Control: How long has their asthma been well-controlled? Generally, asthma should be well-controlled for at least 3 months before considering step-down. Longer periods of control may be preferred in some cases.

6. Patient Understanding and Goals:

Understanding of Step-Down Process: Does the patient understand the rationale for step-down and the potential risks and benefits?

Patient Goals and Concerns: Are there any patient-specific goals or concerns regarding step-down that need to be addressed?

In summary, before stepping down asthma treatment, patients need to demonstrate sustained good asthma control, which includes minimal symptoms, infrequent rescue inhaler use, no exacerbations, good medication adherence and technique, effective trigger management, and a clear understanding of the step-down process. This evaluation is usually done in consultation with a healthcare provider who will assess these factors and determine if step-down is appropriate and safe for the individual patient.

GPT-4o

Before stepping down asthma treatment, patients should be evaluated on the following aspects:

Asthma Control: Patients should have well-controlled asthma with minimal symptoms. This includes no daytime symptoms more than twice a week, no nighttime awakenings due to asthma, and no interference with normal activities.

Frequency of Use of Short-Acting Beta-Agonists (SABA): The patient should use their rescue inhaler (SABA) less than twice a week.

Lung Function: FEV1 (Forced Expiratory Volume in 1 second) and/or peak flow measurements should be stable and at the patient’s personal best or within an acceptable range for their age and health status.

Absence of Exacerbations: There should be no asthma exacerbations requiring oral steroids or emergency treatment in the past few months.

Adherence to Treatment: The patient must be regularly adhering to their prescribed asthma medication regimen.

Comorbidities: Ensure that any comorbid conditions like allergies, obesity, or sinusitis are managed properly, as these can affect asthma control.

Patient’s Preference and Confidence: The patient should be confident in managing their asthma and comfortable with the step-down plan.

This ensures that stepping down treatment is done safely and that asthma remains well-controlled.

Deepseek

Before stepping down asthma treatment, the following factors should be evaluated to ensure safety and maintain control of the condition:

Duration of Asthma Stability

Assess whether asthma has been well-controlled for at least 3–6 months, as shorter stability periods may increase the risk of exacerbation after treatment reduction13611.

Current Level of Asthma Control

Verify that daytime symptoms occur ≤2 times/week, nighttime awakenings are absent, rescue inhaler use is ≤2 times/week, and there are no activity limitations16.

Lung Function

Measure FEV1 (forced expiratory volume in 1 second) and PEF (peak expiratory flow) to confirm stable or improved airway function517.

Risk of Exacerbations

Evaluate recent history of severe attacks, comorbidities (e.g., allergies, sinusitis), and environmental triggers (e.g., smoking, pollution)1418.

Medication Use and Tolerance

Check adherence to the current regimen and prioritize stepping down oral corticosteroids (if used) before reducing inhaled corticosteroids (ICS) or long-acting beta-agonists (LABA)47.

Written Asthma Action Plan

Ensure the patient has a personalized plan to manage worsening symptoms and understands when to seek medical help510.

Patient-Specific Factors

Consider comorbidities, pregnancy, travel plans, or upcoming high-risk periods (e.g., allergy season) that may affect stability1419.

Monitoring and Follow-Up Plan

Schedule regular follow-ups (every 1–6 months) to detect early signs of deterioration post-step-down619.

Stepping down should follow a gradual approach, such as reducing ICS doses first before discontinuing LABAs7. If symptoms recur, revert to the previous treatment level
