# Supplementary material for: Evaluation of large Language models on pediatric asthma: a comparative study of Claude3-Opus, Gemini 2.0, ChatGPT-4o, and DeepSeek—a cross-sectional questionnaire study
Source: BMC Med Inform Decis Mak. 2026 Feb 10;26:77. doi: 10.1186/s12911-026-03371-x (PMC12990414; doi:10.1186/s12911-026-03371-x)
Supplement: Supplementary file 3 — Supplementary Material 3 [file 12911_2026_3371_MOESM3_ESM.docx]

| Claude3-Opus | | DISERN scores | | | | Disern category |
| --- | --- | --- | --- | --- | --- | --- |
|  |  | Specialist1 | Specialist2 | Specialist3 | Mean Scores |  |
| Question1 | What is the preferred initial treatment if a child has infrequent asthma  symptoms.(e.g.1-2days/week or less)? | 53 | 50 | 57 | 53.33 | Good |
| Question2 | If I want to step down asthma treatment,how long should a child asthmatic symptoms be well controlled? | 49 | 46 | 47 | 47.33 | Median |
| Question3 | Can asthma be triggered by strong emotions or will the symptoms be worsened because of the unstable emotions? | 52 | 51 | 48 | 50.33 | Median |
| Question4 | During the asthma attack,do the air tubes collapse?Is More or less mucus produced in the air tubes? | 50 | 51 | 54 | 51.67 | Good |
| Question5 | By listening to the asthma’s patient chest with a stethoscope,Is a doctor manage to measure how bad asthma is? | 49 | 51 | 56 | 52.00 | Good |
| Question6 | What is the treatment option for children whose asthma is not adequately controlled by low-dose maintenance ICS-LABA with as-needed SABA? | 43 | 44 | 49 | 45.33 | Median |
| Question7 | List some add-on biologic therapy for children with uncontrolled severe asthma. | 51 | 53 | 56 | 53.33 | Good |
| Question8 | Will the risk of exacerbation increase when a child switch from Maintenance-and-reliever therapy(MART) to conventional lCS-LABA plus as-needed SABA? | 50 | 47 | 54 | 50.33 | Median |
| Question9 | Will a child be addicted to asthma medications if he uses them frequently? | 41 | 43 | 49 | 44.33 | Median |
| Question10 | After an asthmatic exacerbation,How soon should a review visit be scheduled?What does the frequency of visit depend on? | 35 | 30 | 38 | 34.33 | Bad |
| Question11 | If a child is exposed to viral infections or seasonal allergen exposure,Is a short-term increase in maintenance lCS dose for 1-2 weeks be necessary? | 54 | 53 | 52 | 53.00 | Good |
| Question12 | When an a child with asthma is going to be exposed to something that triggers asthma, can he take medication just before exposure to prevent asthma? | 61 | 60 | 55 | 58.67 | Good |
| Question13 | For children aged 6-11,On what condition can the the treatment be successfully reduced? | 64 | 62 | 60 | 62.00 | Good |
| Question14 | Can high baseline of FeNO been used to predict exacerbation after step-down of lCS dose? | 63 | 65 | 60 | 62.67 | Excellent |
| Question15 | Before asthma treatment step-down,what should be evaluated by patients? | 38 | 36 | 34 | 36.00 | Bad |
|  | Total DISCERN score (16–80) | n=15 |  |  |  |  |
|  | Very bad (16–26) | 0 |  |  |  |  |
|  | Bad (27–38) | 2 |  |  |  |  |
|  | Median (39–50) | 4 |  |  |  |  |
|  | Good (51–62) | 8 |  |  |  |  |
|  | Excellent (63–80) | 1 |  |  |  |  |
|  | Average score | 50.33 ± 8.53 |  |  |  |  |

| Gemini 2.0 | | DISERN scores | | | | Disern category |
| --- | --- | --- | --- | --- | --- | --- |
|  |  | Specialist1 | Specialist2 | Specialist3 | Mean Scores |  |
| Question1 | What is the preferred initial treatment if a child has infrequent asthma  symptoms.(e.g.1-2days/week or less)? | 56 | 54 | 59 | 56.33 | Good |
| Question2 | If I want to step down asthma treatment,how long should a child asthmatic symptoms be well controlled? | 57 | 53 | 60 | 56.67 | Good |
| Question3 | Can asthma be triggered by strong emotions or will the symptoms be worsened because of the unstable emotions? | 52 | 51 | 48 | 50.33 | Median |
| Question4 | During the asthma attack,do the air tubes collapse?Is More or less mucus produced in the air tubes? | 50 | 51 | 54 | 51.67 | Good |
| Question5 | By listening to the asthma’s patient chest with a stethoscope,Is a doctor manage to measure how bad asthma is? | 49 | 51 | 54 | 51.33 | Good |
| Question6 | What is the treatment option for children whose asthma is not adequately controlled by low-dose maintenance ICS-LABA with as-needed SABA? | 43 | 44 | 49 | 45.33 | Median |
| Question7 | List some add-on biologic therapy for children with uncontrolled severe asthma. | 51 | 53 | 56 | 53.33 | Good |
| Question8 | Will the risk of exacerbation increase when a child switch from Maintenance-and-reliever therapy(MART) to conventional lCS-LABA plus as-needed SABA? | 50 | 47 | 54 | 50.33 | Median |
| Question9 | Will a child be addicted to asthma medications if he uses them frequently? | 41 | 43 | 49 | 44.33 | Median |
| Question10 | After an asthmatic exacerbation,How soon should a review visit be scheduled?What does the frequency of visit depend on? | 36 | 32 | 34 | 34.00 | Bad |
| Question11 | If a child is exposed to viral infections or seasonal allergen exposure,Is a short-term increase in maintenance lCS dose for 1-2 weeks be necessary? | 56 | 51 | 57 | 54.67 | Good |
| Question12 | When an a child with asthma is going to be exposed to something that triggers asthma, can he take medication just before exposure to prevent asthma? | 60 | 63 | 57 | 60.00 | Good |
| Question13 | For children aged 6-11,On what condition can the the treatment be successfully reduced? | 66 | 67 | 61 | 64.67 | Excellent |
| Question14 | Can high baseline of FeNO been used to predict exacerbation after step-down of lCS dose? | 63 | 65 | 62 | 63.33 | Excellent |
| Question15 | Before asthma treatment step-down,what should be evaluated by patients? | 38 | 35 | 39 | 37.33 | Bad |
|  | Total DISCERN score (16–80) | n=15 |  |  |  |  |
|  | Very bad (16–26) | 0 |  |  |  |  |
|  | Bad (27–38) | 2 |  |  |  |  |
|  | Median (39–50) | 4 |  |  |  |  |
|  | Good (51–62) | 7 |  |  |  |  |
|  | Excellent (63–80) | 2 |  |  |  |  |
|  | Average score | 51.60 ± 9.04 |  |  |  |  |

| ChatGPT-4o | | DISERN scores | | | | Disern category |
| --- | --- | --- | --- | --- | --- | --- |
|  |  | Specialist1 | Specialist2 | Specialist3 | Mean Scores |  |
| Question1 | What is the preferred initial treatment if a child has infrequent asthma  symptoms.(e.g.1-2days/week or less)? | 58 | 53 | 62 | 57.67 | Good |
| Question2 | If I want to step down asthma treatment,how long should a child asthmatic symptoms be well controlled? | 56 | 54 | 59 | 56.33 | Good |
| Question3 | Can asthma be triggered by strong emotions or will the symptoms be worsened because of the unstable emotions? | 55 | 54 | 51 | 53.33 | Good |
| Question4 | During the asthma attack,do the air tubes collapse?Is More or less mucus produced in the air tubes? | 52 | 50 | 56 | 52.67 | Good |
| Question5 | By listening to the asthma’s patient chest with a stethoscope,Is a doctor manage to measure how bad asthma is? | 53 | 51 | 53 | 52.33 | Good |
| Question6 | What is the treatment option for children whose asthma is not adequately controlled by low-dose maintenance ICS-LABA with as-needed SABA? | 45 | 49 | 50 | 48.00 | Median |
| Question7 | List some add-on biologic therapy for children with uncontrolled severe asthma. | 49 | 52 | 51 | 50.67 | Good |
| Question8 | Will the risk of exacerbation increase when a child switch from Maintenance-and-reliever therapy(MART) to conventional lCS-LABA plus as-needed SABA? | 49 | 48 | 52 | 49.67 | Median |
| Question9 | Will a child be addicted to asthma medications if he uses them frequently? | 46 | 44 | 48 | 46.00 | Median |
| Question10 | After an asthmatic exacerbation,How soon should a review visit be scheduled?What does the frequency of visit depend on? | 40 | 41 | 39 | 40.00 | Median |
| Question11 | If a child is exposed to viral infections or seasonal allergen exposure,Is a short-term increase in maintenance lCS dose for 1-2 weeks be necessary? | 55 | 52 | 50 | 52.33 | Good |
| Question12 | When an a child with asthma is going to be exposed to something that triggers asthma, can he take medication just before exposure to prevent asthma? | 60 | 58 | 59 | 59.00 | Good |
| Question13 | For children aged 6-11,On what condition can the the treatment be successfully reduced? | 63 | 61 | 57 | 60.33 | Good |
| Question14 | Can high baseline of FeNO been used to predict exacerbation after step-down of lCS dose? | 62 | 66 | 62 | 63.33 | Excellent |
| Question15 | Before asthma treatment step-down,what should be evaluated by patients? | 38 | 36 | 34 | 36.00 | Bad |
|  | Total DISCERN score (16–80) | n=15 |  |  |  |  |
|  | Very bad (16–26) | 0 |  |  |  |  |
|  | Bad (27–38) | 1 |  |  |  |  |
|  | Median (39–50) | 4 |  |  |  |  |
|  | Good (51–62) | 9 |  |  |  |  |
|  | Excellent (63–80) | 1 |  |  |  |  |
|  | Average score | 51.87±7.65 |  |  |  |  |

| DeepSeek | | DISERN scores | | | | Disern category |
| --- | --- | --- | --- | --- | --- | --- |
|  |  | Specialist1 | Specialist2 | Specialist3 | Mean Scores |  |
| Question1 | What is the preferred initial treatment if a child has infrequent asthma  symptoms.(e.g.1-2days/week or less)? | 51 | 47 | 53 | 50.33 | Median |
| Question2 | If I want to step down asthma treatment,how long should a child asthmatic symptoms be well controlled? | 46 | 49 | 45 | 46.67 | Median |
| Question3 | Can asthma be triggered by strong emotions or will the symptoms be worsened because of the unstable emotions? | 47 | 50 | 52 | 49.67 | Median |
| Question4 | During the asthma attack,do the air tubes collapse?Is More or less mucus produced in the air tubes? | 50 | 55 | 56 | 53.67 | Good |
| Question5 | By listening to the asthma’s patient chest with a stethoscope,Is a doctor manage to measure how bad asthma is? | 50 | 52 | 54 | 52.00 | Good |
| Question6 | What is the treatment option for children whose asthma is not adequately controlled by low-dose maintenance ICS-LABA with as-needed SABA? | 44 | 45 | 50 | 46.33 | Median |
| Question7 | List some add-on biologic therapy for children with uncontrolled severe asthma. | 54 | 49 | 52 | 51.67 | Good |
| Question8 | Will the risk of exacerbation increase when a child switch from Maintenance-and-reliever therapy(MART) to conventional lCS-LABA plus as-needed SABA? | 46 | 47 | 54 | 49.00 | Median |
| Question9 | Will a child be addicted to asthma medications if he uses them frequently? | 40 | 42 | 46 | 42.67 | Median |
| Question10 | After an asthmatic exacerbation,How soon should a review visit be scheduled?What does the frequency of visit depend on? | 41 | 42 | 44 | 42.33 | Median |
| Question11 | If a child is exposed to viral infections or seasonal allergen exposure,Is a short-term increase in maintenance lCS dose for 1-2 weeks be necessary? | 54 | 53 | 52 | 53.00 | Good |
| Question12 | When an a child with asthma is going to be exposed to something that triggers asthma, can he take medication just before exposure to prevent asthma? | 62 | 61 | 59 | 60.67 | Good |
| Question13 | For children aged 6-11,On what condition can the the treatment be successfully reduced? | 64 | 62 | 60 | 62.00 | Good |
| Question14 | Can high baseline of FeNO been used to predict exacerbation after step-down of lCS dose? | 55 | 57 | 58 | 56.67 | Good |
| Question15 | Before asthma treatment step-down,what should be evaluated by patients? | 40 | 44 | 42 | 42.00 | Median |
|  | Total DISCERN score (16–80) | n=15 |  |  |  |  |
|  | Very bad (16–26) | 0 |  |  |  |  |
|  | Bad (27–38) | 0 |  |  |  |  |
|  | Median (39–50) | 8 |  |  |  |  |
|  | Good (51–62) | 7 |  |  |  |  |
|  | Excellent (63–80) | 0 |  |  |  |  |
|  | Average score | 50.57 ± 6.40 |  |  |  |  |
